# Community detection in sequence similarity networks based on attribute clustering

**DOI:** 10.1371/journal.pone.0178650

**Published:** 2017-07-24

**Authors:** Janamejaya Chowdhary, Frank E. Löffler, Jeremy C. Smith

**Affiliations:** 1 Center for Molecular Biophysics, Oak Ridge National Laboratory, Oak Ridge, Tennessee, United States of America; 2 University of Tennessee-Oak Ridge National Laboratory, Joint Institute for Biological Sciences and Biosciences Division, Oak Ridge National Laboratory, Oak Ridge, Tennessee, United States of America; 3 Department of Microbiology, Department of Civil and Environmental Engineering, University of Tennessee, Knoxville, Tennessee, United States of America; 4 Center for Environmental Biotechnology, University of Tennessee, Knoxville, Tennessee, United States of America; 5 Department of Biochemistry and Cellular and Molecular Biology, University of Tennessee, Knoxville, Tennessee, United States of America; Universidad Rey Juan Carlos, SPAIN

## Abstract

Networks are powerful tools for the presentation and analysis of interactions in multi-component systems. A commonly studied mesoscopic feature of networks is their community structure, which arises from grouping together similar nodes into one community and dissimilar nodes into separate communities. Here, the community structure of protein sequence similarity networks is determined with a new method: Attribute Clustering Dependent Communities (ACDC). Sequence similarity has hitherto typically been quantified by the alignment score or its expectation value. However, pair alignments with the same score or expectation value cannot thus be differentiated. To overcome this deficiency, the method constructs, for pair alignments, an extended alignment metric, the link attribute vector, which includes the score and other alignment characteristics. Rescaling components of the attribute vectors qualitatively identifies a systematic variation of sequence similarity within protein superfamilies. The problem of community detection is then mapped to clustering the link attribute vectors, selection of an optimal subset of links and community structure refinement based on the partition density of the network. ACDC-predicted communities are found to be in good agreement with gold standard sequence databases for which the “ground truth” community structures (or families) are known. ACDC is therefore a community detection method for sequence similarity networks based entirely on pair similarity information. A serial implementation of ACDC is available from https://cmb.ornl.gov/resources/developments

## 1. Introduction

A core objective of bioinformatics is classification of sequences. Given a set of sequences, each sequence is assigned to a sequence family which corresponds to evolutionarily related sequences sharing significant sequence, structure, and/or mechanistic similarity. Sequence families sharing overall structural and/or mechanistic similarity suggestive of a common evolutionary origin are grouped together into a superfamily. As only a tiny fraction of known sequences have been experimentally characterized, such a classification of sequences is important for developing, for example, functional hypotheses [1], identification of erroneous functional annotation [2], and understanding evolutionary mechanisms underlying enzyme sequence variation within superfamilies [3]. Due to availability of millions of protein and gene sequences due to affordable sequencing, it is imperative that accurate classification (e.g., [4]) of sequences be performed for making sense of the sequence universe.

Membership of a sequence in a sequence family is based on similarity between pairs of sequences. Sequence similarity is typically quantified using pair sequence alignments. For a given amino-acid or nucleotide substitution matrix and associated gap open and gap extension penalties, an objective function is defined which assigns a cost for identical and mismatched residues based on the substitution matrix, and a penalty for introducing and extending gaps. During pair sequence alignment, this objective function is maximized and an optimal gapped alignment is identified [5]. Such an alignment is fully quantified by the alignment length (*L*_a_), the number of identical residues (*N*_id_), the number of mismatched residues (*N*_m_) and the number of gaps (*N*_g_). The problem of finding an optimal alignment is equivalent to finding the ground state of a directed path in a 2-dimensional random medium [6] to which the optimal alignment corresponds to the ground state of the directed path and the alignment score (*S*) is then analogous to the negative free energy of the ground state [7]. The statistical significance of a score is quantified in terms of an expectation value (E-val) and measures the excess similarity for the pair alignment relative to alignment with random sequences [8, 9]. These alignment attributes then fully quantify similarity between sequence pairs.

A convenient framework for simultaneous incorporation of all pair similarity information is that of networks. A network is a visually efficient and mathematically convenient representation of information contained in a relational system [10, 11]. In general, a network can be represented as an undirected attributed graph *G* = {*V*, *E*, *A*_V_, *A*_E_} which, at a minimum, contains as its elements the set *V* of *N*_V_ nodes or vertices and a set *E* of *N*_E_ links or edges. Each node *i* in set V corresponds to an indivisible interacting component of the system and each link in set *E* connects a pair of nodes depending of the presence of some relationship. The topology of a network is completely specified by the set of nodes and links. Additionally, each node may be associated with a set of attributes or metadata, e.g., the name and age of individuals in a social network. Similarly, each link may be associated with attributes, e.g., shared hobbies between two individuals in a social network. With the set of node and link attributes, *A*_v_ and *A*_E_, respectively, all information in a network is completely specified. In general, a network’s topology and attributes are correlated [12]. When combined with the availability of network data from different sources and the rapid development of tools for their analysis, network analysis has become a cornerstone of machine learning and data mining with applications in diverse research areas, involving such as social [13], biological [14], and spatial [15] networks.

One of the objectives of network analysis is to reveal the local, mesoscopic, and global relationships in the data. At the local level, network structure can be characterized, say, in terms of the degree of a node, which corresponds to the number of links involving the node. At the mesoscale, a commonly studied feature is that of communities [16–18], which can be interpreted as sets of nodes or links with more similarity between them than with other nodes or links in the network. For example, a social network with links arising solely due to shared hobbies between individuals may contain communities of individuals sharing a common hobby. The global structure of the network contains information on distributions of network characteristics such as the degree. Based on suitable measures of network structure at different scales, data mining can lead to valuable information on the important elements and interactions within the network.

In the context of sequence classification, a sequence similarity networks (SSNs) [19, 20] represents each sequence as a node in a network and each pair of nodes is connected with an undirected link representative of the similarity between them. The similarity between any two sequences in the SSN can be incorporated as the link attribute vector based on a suitable choice of similarity attributes. Due to their ability to simultaneously incorporate and present sequence similarity information for multiple sequences, SSNs have become an important tool for sequence classification [21–26]. The hypotheses behind the use of SSNs for sequence classification is that communities in the network correspond to sequence “families” with more significant intra-community than inter-community sequence similarity and that these communities are separable.

As each node in an SSN participates in *N*_v_-1 links, the local network structure is identical for each node if link and node attributes are ignored. So, community detection methods that depend on the local structure around each node will fail [16]. An alternative approach is link community detection [27]. In this approach, node communities are defined as groups of similar or related links based on the line graph of the network, i.e, a graph where each node corresponds to a link in the original network [28]. However, all links in an SSN are topologically equivalent. Hence, communities cannot be identified based solely on network topology when link or node attributes are ignored.

Most methods for detecting communities in SSNs make the reasonable assumption that the score or E-val are good measures of sequence similarity and use them as edge attributes for the network. The inclusion of weights breaks the local symmetry of the SSN and makes it possible to analyze it. However, this progress comes with a cost. In order to identify communities, SSN analysis methods typically require a user specified cutoff value for these edge metrics or cutoff values for some method-dependent parameters. With these assumptions and parameters in place, communities can be detected based on approaches such as cut-based identification of connected components [19, 20, 29] where only edges with weights exceeding a single cutoff value of the score or expectation value, applicable to all links, are retained in the network. Alternative methods that depend on flow/random walks on the network (MCL [30]), or network geometry (spectral clustering [31] and weighted graph cluster editing TransClust [32]) are available. Such methods can lead to communities for which the smallest edge weight within the community differs, unlike a fixed value in a single-cut based approach, and this represents a more general strategy for community detection. Not surprisingly, different methods emphasize different aspects of the network and often lead to differences in community membership of sequences [33].

Here we propose a novel method, Attribute Clustering Dependent Communities (ACDC), for community structure detection in protein SSN which utilizes attribute vector clustering and excludes user-tunable parameters. First, new link attribute vectors with pair alignment information as their components are proposed. Rescaling components of **a** link attribute vector reveals a novel systematic variation of sequence similarity within protein superfamilies. Second, the distribution of attribute vectors in space is analyzed in order to identify the number of clusters. Links in the identified clusters are filtered in a systematic manner such that an objective function that quantifies network structure, the link partition density [27], is maximized in order to identify and refine the community structure of the SSN. The use in ACDC of partition density maximization eliminates the need for any user defined cutoff, unlike most methods for network clustering which do need have tunable user selected parameter(s). Third, in order to provide a reference best performance community structure, multidimensional grid searches are performed to identify optimal cutoff attribute vectors that best reproduce the “ground truth” community structure for selected superfamilies. A key outcome of the analysis is the identification of the topology of attribute space and key features relevant to community detection. The method is applied successfully to gold standard datasets for which “ground truth” community structure is known.

## 2. Methods

### 2.1. Link attribute selection for community detection

The SSN for a set of sequences is based entirely on pair alignments between them. The E-val for a pair alignment is calculated based on comparison of its score *S* with scores for sequence alignments between randomly generated sequences. Such random sequences are external to the sequences in the SSN and consequently, the E-val is considered an extrinsic measure of similarity. Here, we discard such extrinsic measures. By comparison, the alignment score, *S*, is calculated for each pair of sequences in the network and is an intrinsic network attribute. It should be noted that *S* and E-val are closely related; small E-vals tend to be associated with large values of *S*. Typically most alignment programs convert scores to bit-scores. In the following bit-scores will be referred to as the score.

Two pair alignments with very similar or identical values of *S* need not have the same set of values for *L*_a_, *N*_id_, *N*_m_, and *N*_g_ (S1 Table). Thus, although score is a useful quantity, additional information is necessary in order to differentiate between these two pair alignments. Here, it is proposed that the score be supplemented with three other alignment attributes, i.e., *L*_a_, *N*_id_, and *N*_m_. Since *L*_a_ = *N*_id_ + *N*_m_ + *N*_g_, any two attributes on the right side of the equation can be selected as independent variables. Without any loss of information, *N*_g_ can be ignored. Now, pair alignments with very similar scores should be differentiateable based on their attribute vectors (*L*_a_, *N*_id_, *N*_m_, *S*).

Each attribute vector embodies the function *S* = S(*L*_a_, *N*_id_, *N*_m_) and the magnitude of *S* depends parametrically on the substitution matrix. In order to identify universal features of a multivariate function, it is often useful to scale the variables by some natural scale factor implicit in the system and consider the functional variation after scaling. This is particularly useful for free energy functions, such as the alignment score[7]. Furthermore, the magnitude of individual variables is typically different and normalizing the data is a good practice. To incorporate these potential benefits, scale factors will be presented in the following and the resultant scaled attribute vector is presented.

For the pair alignment of two sequences, A and B, each with identical sequence lengths, the maximum value of *S* (= *S*_max_) is obtained when A and B are identical. As sequence B diverges from A, *S* is expected to decrease. Thus, *S* takes on values from 0 to *S*_max_, and *S*_max_ is a reasonable scale factor. In the case of distinct sequence lengths, the largest score is obtained when one of the sequences is a subsequence of the other. The score, *S*_min_, then corresponds to the self-alignment score for the shorter sequence or the smaller self-alignment score, if the shorter sequence does not have the smaller self-alignment score. With *S*_min_ as an appropriate scale factor, the scaled variable, *s*_a_ = *S*/*S*_min_, then varies between 0 and ≈1, with the largest value depending on the amino acid composition and the substitution matrix.

Similarly, for two sequences A and B with different sequence lengths, *L*_a_ is typically equal to or less than the length of the smaller sequence, *L*_min_, although some values of *L*_a_ may exceed *L*_min_ when gaps are present. Thus, *L*_min_, is an appropriate scale factor and the scaled alignment length, *l*_a_ = *L*_a_/*L*_min_, varies between 0 and ≈1. Scaled values for the remaining two attributes, *N*_id_ and *N*_m_, can be obtained by scaling them by *L*_a_. Now they represent the fraction of identical residues, *f*_id_, and the fraction of mismatched residues, *f*_m_, of the alignment length. The rescaled set of link attributes is now defined as A_E,S_ = {(*l*_a_, *f*_id_, *f*_m_, *s*_a_)_(i)_} where (i) represents the *i*^th^ links attribute vector. A useful consequence of this scaling is that any distance between points in attribute space is not dominated by the variation of a numerically large attribute[34]. It should be noted that the scaling proposed here is by no means the only possibility but is nevertheless clearly based on naturally existing scales.

For a perfect alignment, the scaled attribute vector is (1, 1, 0, 1). The deviation of any pair alignment from this reference perfect alignment can be quantified in terms of the Euclidean distance, *d*, between the two attribute vectors which can take values between 0 (for a perfect alignment) and ≈2 (for the worst possible alignment). Thus, *d* is a measure of the evolutionary distance between pairs of sequences.

### 2.2. Cut based grid search for community detection (GridS)

Cut-based methods are among the simplest methods for community detection in weighted networks. The main idea is to partition the network by selecting all link weights that exceed a single preselected cutoff value. For a network where each link is associated with a link-attribute vectors, instead of a scalar weight, a cutoff value for each link attribute vector component can be combined to construct a cutoff link attribute vector, A_E,c_ = (*l*_a,c_, *f*_id,c_, *f*_m,c_, *s*_a,c_), where the subscript c stands for cutoff value. The set of attribute vectors is then partitioned based on A_E,c_ such that only links for which *l*_a_≥*l*_a,c_, *f*_id_≥*f*_id,c_, *f*_m_≤*f*_m,c_, and *s*_a_≥*s*_a,c_ are retained. Nodes can be connected based on the retained links and naturally group together into communities. An optimal choice of A_E,c_ is such that the resulting communities are in best agreement with the “ground truth” community structure of the network.

A simple brute force search method for identifying an optimal {A_E,c_} is by (a) varying cutoff attribute values along a four multidimensional grid, (b) identifying links corresponding to each cutoff attribute vector, (c) connecting the set of identified links in order to assemble communities in the network, (d) quantifying the agreement of the derived community structure with “ground truth” community structure based on some measure, *Q*, which will be defined later, and (e) selecting the maximum value of *Q*, *Q*_max,n_ where *n* is the dimensionality of the space being searched. This method will be referred to as GridS in the following

### 2.3. Attribute clustering dependent communities (ACDC)

#### 2.3.1. Community detection strategy

Inspired by a cut-based connected component approach to community detection, we propose the following hypotheses:

1. Alignments between sequence pairs with significant sequence similarity (the related sequences) and those between sequence pairs with insignificant sequence similarity (the unrelated sequences) occupy topologically distinct regions in attribute space.
2. The two distinct regions are connected with a transition region in attribute space containing continuously varying attribute vectors.
3. There exists a separatrix that partitions attribute space into two distinct regions, one containing sequence pairs with significant sequence similarity and the other containing the remaining attribute vectors.
4. There exists a function whose maximum value is achieved for the “correct” community structure for the network.

In hypothesis 1, sequence pairs with high sequence similarity implies that they either share common ancestry, i.e., they are homologs, or they have different ancestral sequences but are still similar, i.e. they are analogs. Highly similar sequence pairs tend to have large *S*, *L*_a_ and *N*_id_, and small *N*_m_ values. Unrelated sequences, on the other hand, have alignments with small percentage identities arising from pairs of random sequences or very distant related sequences. Such unrelated sequence pairs tend to have small *S*, *L*_a_, and *N*_id_, and large *N*_m_ values. Clearly, these two types of attribute vectors (or their scaled versions) are likely to occupy distinct regions of attribute space and the hypothesis is reasonable.

It is well known that each alignment attribute contributing to the link attribute vector is a continuous variable[35], and hence hypothesis 2 is also reasonable. For a given protein superfamily, the distribution of attribute values may be discrete but that is only a result of the finiteness of the sequence dataset.

Hypothesis 3 is the equivalent of the cut-based connected approach to community detection formulated in terms of attribute space. In the case of a weighted network using *S* as the link weight, a cutoff value of the link weight is selected such that all links with weights lower than the cutoff value are excluded. By analogy, it is proposed that there exists a separatrix passing through the transition region which separates attribute space into regions containing pair alignments between related or unrelated sequences.

Hypothesis 4 states a general approach towards community detection according to which there exists an objective function that implicitly or explicitly incorporates some property of community structure such that its extrema correspond to a good community structure of the network [36, 37]. A number of quality functions exist, each optimal for specific contexts. An ideal feature of these methods is that a uniform cutoff scheme, as explicit in the Separatrix of Hypothesis 3, is now replaced with an implicit variable cutoff scheme where each community can have a different cutoff attribute vector. Thus, more variability of attribute vectors within communities, independent of other communities, can be accommodated.

#### 2.3.2. Clustering and objective function maximization for community detection

In the context of an SSN, a community of nodes represents a set of sequences that are more similar to each other (i.e., homologous) than to sequences from a different community. Consider two sequences A and B. If A and B belong to different communities, they are expected to have a much smaller measure of sequence similarity than when they belong to the same community. In this case, all pairs of sequences within a community are more similar than all pairs of sequences from distinct communities. Thus, intra-community pair similarities and inter-community pair similarities define two limiting regimes of sequence similarity.

For communities with more than two nodes, any three, or more, nodes, connected together with links, can be related through transitive homology. As per the transitive property of homology, if sequence A is homologous to sequence B, and sequence B is homologous to sequence C, then sequences A and C are also homologous, even if they do not share significant sequence similarity. If within a community, sequences A and C have diverged sufficiently, their sequence similarity may be indistinguishable from inter-community sequence similarity. On the other hand, they may be sufficiently similar to be indistinguishable from intra-community pair sequence similarity. Thus, all sequences related through transitive homology have pair alignment similarity measures that span a broad range of values intermediate to the two limiting sequence similarity measures for inter- and intra-community pair similarity.

Consider the three sequences A, B and C within a community. Provided the similarity cutoff is large enough to include the most distant homologous sequence pairs in the community, as long as the links between A and B, and B and C are included, the transitive homology relation between them will always place A, B and C within the same community. Thus, a minimal set of links associated with large pair sequence similarity attributes should be sufficient for community detection. Here, it is proposed that in scaled pair alignment attribute space, all such pair attributes represent points that should occupy the same high sequence similarity part of attribute space, and that this is approximately separable from all other similarity attributes. Based on this hypothesis, such a region could be identifiable based on the unsupervised learning method of clustering. The problem of community detection in SSNs is now converted in the ACDC method into the detection of clusters of points in link-attribute space and selecting the cluster with attribute values corresponding to high sequence similarity.

Since a clearly defined hypersurface separating intra- from inter-community links or between clusters, may not exist, it is likely that several links that contribute to the high sequence similarity cluster will need to be excluded in the community detection. The identification of an optimal set of links based on their attribute vectors could be implemented either as a simple multidimensional-cut based method that would select only the links for which attribute values satisfy some constraint. Alternatively, this objective could be implemented as a functional optimization in which the subset of links is selected such that a function that quantifies the community structure of the network based on attribute values is optimized. Unfortunately, for most real networks with known ground truth community structures, no single optimal quality measure of the networks community structure exists[36]. The search for quality measures whose optimal value represents a good community structure is an area of ongoing research [37].

The overall structure of a network community is defined by the number of nodes in it and the distribution of links between them. The most commonly used objective function for community structure optimization is Modularity[16]. Modularity is a measure of how compact the arrangement of nodes in a network community is, as compared to a network with randomly assigned links between the same set of nodes, while keeping the order for each node unchanged. Alternatively, the density of nodes in a community can be quantified by Partition Density[27] which compares the distribution of links within a community with reference structures that correspond to limiting cases of link connectivity, i.e., a chain of links versus a maximally connected clique. Due to its clearer structural interpretation and better performance in link based community detection[27], partition density was selected as the objective function for community structure optimization. For a network with *N* links and a partition of these links {P_1_, P_2_, …, P_C_} into C subsets, let subset P_C_ have *m*_C_ links and *n*_C_ nodes. Then, the partition density *D* is defined as

$$D=\frac{2}{N}\Sigma_{C}m_{C}\frac{m_{c}-\left(n_{C}-1\right)}{\left(n_{C}-2\right)\left(n_{C}-1\right)}$$ (1)

The summation is limited to communities with more than two nodes in them. Note that for a single community network, in the limit of a linearly connected chain of nodes, *m*_c_ = *n*_c_-1 and *D* is zero. In the limit of a fully connected single community network (as for the unfiltered SNN), *m*_c_ = *n*_c_(*n*_c_-1)/2 and *D* is unity. The partition density prefers maximal inter-node connectivity in communities, although this tendency is limited by averaging over all communities.

#### 2.3.3. Community structure refinement

Due to the absence of a universal objective function whose optimization leads to an optimal community structure prediction for the network, any resulting solution is likely to contain communities with erroneously assigned links. In this scenario, there is a need for a procedure for refining the community structure based on the scaled attribute vectors.

While the potential number of errors in community structure prediction is *a priori* unknown, at least two types of error can be anticipated. By the consensus definition of a community, similarity between nodes within a community should exceed similarity between nodes between distinct communities. One possible error arises if this simple criterion is not satisfied. In the following, this error will be referred to as a Type 1 error. Let Z correspond to the similarity measure between two nodes connected by a link and let small values of Z imply highly similar nodes. Given the simple definition of Type 1 errors, the following procedure can be used to account for this error. Let *Z*_max_(*i*,*i*) and *Z*_max_(*j*,*j*) be the largest values of some pre-selected intra-community similarity measure between pairs of nodes in communities *i* and *j*, respectively. Let Z_min_(i,j) be the smallest value of the inter-community similarity measure between communities *i* and *j*. Then, the presence of a Type 1 error implies that the condition C_1_: Z_min_(i,j)≤max[Z_max_(i,i),Z_max_(j,j)] is not satisfied. In this case, the solution is to simply merge communities *i* and *j*. The set of inter-community links to be added to the merged community should ensure that condition C_1_ is strictly enforced.

A second type of error may arise if a single “ground truth” community is split into two communities during optimization of the objective function such that condition C_1_ is satisfied. Such an error will be referred to as a Type 2 error in the following. A Type 2 error indicates that the optimization converged on a solution for which the final *Z*_max_(*i*,*i*) values are smaller than would be required for the two communities to be merged automatically. Alternatively, this may indicate that the link required to merge the split communities does not exist. A solution to Type 2 error is to override condition C_1_ and include links with *Z* values up to some preset value, *Z*_0_. In this case, the value of *Z*_0_ should be small enough that this correction does not make the merged community eligible for merger with some other community for which links satisfying C_1_ exist. If such a situation arises, the split communities are not merged.

For a network with *M* communities resulting from function optimization, the following implementation of a community refinement procedure to account for Type1 and Type 2 errors is proposed: Select a scale factor, *s*_f_>1, and a maximum value of the similarity measure, *Z*_0_.

1. Let *M* be the number of communities. Construct the *M*x*M* matrices *Z*_max_ and *Z*_min_
2. Identify the largest value of *Z*, *Z*_m_, from the set of intra-community links.
3. communities i≠j, if C_1_ is false, tag the community pairs and go to Step 4, else Step 5.
4. Merge communities *i*≠*j*. Add links between communities *i* and *j* for which the similarity measure *Z*≤max[Z_max_(i,i),Z_max_(j,j)].
5. Let *N* be the new number of communities. Construct *N*x*N* matrices *Z*_max_ and *Z*_min._
6. *Z*_m_ ←*s*_f_ *Z*_m_
7. Find left outliers, *Z*_L_, from all values of *Z*_min_.
8. communities *i*≠*j*, if *Z*≤min{*Z*_L_, *Z*_m_, *Z*_0_}, merge communities *i* and *j* with those links. If no new links found, go to Step 11.
9. Evaluate *Z*_m_.
10. If *Z*_m_≥*Z*_0_, go to Step 11, else, go to Step 1
11. End

The proposed method starts by testing for Type 1 errors and merging communities, as required. Next, it considers Type 2 errors. Based on the hypothesis that split communities are likely to have inter-community *Z*_min_ values that corresponds to a process distinct from the process of formation of two distinct communities, the former is expected to be an outlier in the distribution of the inter-community similarity measure values. Starting with a given community structure, the proposed method searches for pairs of communities for which *Z*_min_(i,j)≤*Z*_L,_ where *Z*_L_ is the lower outlier bound, and merges pairs of communities that are so identified. In the case Z_L_≤Z_m_, the analysis reduces to that for Type 1 errors. If *Z*_L_≥*Z*_m_, the score of the search and merger is extended by scaling *Z*_m_ by the factor *f*_s_≥1.

As a systematic procedure, a two-dimensional grid in the (s_f_, Z_0_) plane is considered. For each grid point, the procedure outlined in the previous paragraph is followed and the partition density evaluated for the resultant community structure. The grid point that leads to the largest value of partition density is selected and the corresponding community structure is selected as the final community structure for the network. While this refinement procedure is expected to improve the agreement with the “ground truth” community structure, it may still not lead to elimination of all split communities.

A third type of error may arise when two “ground truth” communities are merged during optimization of the objective function. While it is tempting to consider such merged communities as equivalent to overlapping communities, the presence of additional links between them may complicate the splitting of such merged communities. Such splitting of merged communities will not be considered here.

### 2.4. Data sets, sequence similarity network construction, and visualization

The gold standard (GS) protein sequence dataset of Brown *et al*. [38] with known “ground truth” community structure was selected for sequence similarity network construction. This dataset consists of 866 protein sequences from 91 families (or communities) that belong to five mechanistically diverse enzyme superfamilies. The classification into communities is based on experimental information on the reactions catalyzed by these enzymes. The GS dataset is considered in its entirety as well as split into five subsets, each consisting of a single constituent superfamily from the GS dataset. The number of sequences and communities in each superfamily are summarized in Table 1. Validating ACDC against these six GS datasets then tests its ability to distinguish between families within superfamilies as well as in a mixture of superfamilies. Recently, NCBI has replaced GI numbers with the corresponding Accession number to refer to individual sequences. As the Gold Standard sequence dataset identifies sequences by the GI number, we have included the mapping of each GI number to the corresponding NCBI accession number as S2 Table.

**Table 1:** Sequence datasets: Network properties, number of communities, and their use in method development.

Dataset	#Sequences	#Links	#Communities	Usage
**Amidohydrolase**	*232*	*26796*	*29*	*Benchmark*
**Crotonase**	*91*	*4095*	*16*	*Validation*
**Enolase**	*285*	*40470*	*9*	*Validation*
**Haloacid Dehalogenase**	*125*	*7750*	*20*	*Validation*
**Vicinyl Oxygen Chelatase**	*113*	*6328*	*17*	*Benchmark*
**Gold Standard**	*866*	*374545*	*91*	*Validation*

In order to identify the region of attribute space occupied by potential evolutionarily unrelated sequences with insignificant sequence similarity, 10000 randomly generated sequences, each with 100 residues, were constructed. Using HMMscan in the HMMER3 suite of programs [39], all sequences were searched against the library of Pfam [22] profile hidden Markov models for potential domains using the default gathering threshold for each profile hidden Markov model. Sequences with any domain matches were removed. For the remaining sequences, an all-by-all pair sequence comparison was performed. Pairs of sequences that share at most 15–20% identity over less than 75 residues were identified. Only 19 sequences were found and these constitute the R15 dataset. Given the small percentage identity in R15, they are expected to be unrelated or at least very distantly related. As a result, it is reasonable to assume that each of these sequences constitutes a separate community/protein family.

The numbers of sequences and families in each superfamily from the selected dataset are listed in Table 1. The Amidohydrolase (AH) and Vicinyl Oxygen Chelatase (VOC) superfamily were selected at random from the GS dataset for learning the structure of attribute space and benchmarking the community detection method. The remaining datasets were used to perform the actual evaluation of the community detection method.

An SSN was constructed for each dataset by using each sequence in the dataset as a query sequence and all sequences in the dataset as the target database. The sequence similarity attributes were calculated with the Smith-Waterman [40] method for optimal alignment detection as implemented in the SSEARCH program that is part of the FASTA suite of programs [41]. The default amino-acid substitution matrix used by SSEARCH, BLOSUM50, was selected along with the default gap open and extension penalties. Since protein sequences often contain repeats and composition biases, the program segmasker in the NCBI BLAST suite [9] was used to mask these regions in all protein sequences prior to their pair alignment. For each pair of sequences in the dataset, the pair alignment information was stored in BLAST tabular format. In general, for a pair of sequences A and B, selection of A as the query and B as the library sequence during pair alignment typically leads to a different alignment from that of B as the query and A as the library sequence. To account for this asymmetry, the highest scoring pair alignment was selected to represent the pair. In this manner, all pair alignments were collected for each protein superfamily.

Alignment metrics for all pair alignments in each selected groups of clusters were collated into a tab separated table. The network table was imported into Cytoscape 3.2.1 [42], a versatile network visualization and analysis application. The network was visualized with the Yfiles-Organic Layout, which has been demonstrated to group nodes connected by edges representing large percentage identities close together in space [19].

### 2.5. Data processing for community detection

#### 2.5.1. Cut based grid search

An important factor in grid searches is the increment in each component value between neighboring grid points. For a fine-grained grid, performing a full grid search calculation is computationally prohibitive. On the other hand, a coarse-grained grid may miss important set of cutoffs. A compromise used here is to use a fine grid with a spacing of 0.01 along three dimensional subspaces, selecting a subset of best performing grid points, and then extending the grid search with a spacing of 0.01 along the fourth dimension.

Since the attribute space is four dimensional, all combinations of three dimensional subspaces {(*l*_a_, *f*_id_, *f*_m_), (*l*_a_, *f*_id_, *s*_a_), (*l*_a_, *f*_m_, *s*_a_), (*s*_a_, *f*_id_, *f*_m_)} are selected. Grid searches are performed and *Q* is calculated for all cutoff attribute vectors {(*l*_a,c_, *f*_id,c_, *f*_m,c_), (*l*_a,c_, *f*_id,c_, *s*_a,c_), (*l*_a,c_, *f*_m,c_, *s*_a,c_), (*s*_a,c_, *f*_id,c_, *f*_m,c_)}. The largest value of *Q* from the subspace search, *Q*_max,3_, is identified and the set of cutoff attribute vectors for which *Q* > = 0.9 *Q*_max,3_ is shortlisted. The scale factor of 0.90 is selected in order to limit the fourth dimensional search to the region most likely to contain large or larger values of *Q*. For each shortlisted cutoff attribute vector, a one-dimensional scan of the missing fourth dimension is performed using a grid spacing of 0.01. Only cutoff attribute vectors for which *Q*≥0.90*Q*_max,3_ are saved. These cutoff attribute vectors approximately represent an optimal hypersurface which brackets the part of attribute space required for optimal community detection. Knowledge of this hypersurface as well as *Q*_max,4_ values provides a useful reference for comparing the performance of community detection methods.

#### 2.5.2. Attribute clustering dependent communities

To identify clusters in attribute space, the implementation of k-means clustering in MATLAB (R2015a, The MathWorks Inc., Natick, MA, 2000) was selected for cluster detection. The optimal number of clusters in attribute space for each superfamily is *a priori* unknown. Since higher-dimensional attribute vectors cannot be visualized easily, Principal Component Analysis (PCA) [43] is performed using MATLAB to identify linear combinations of attribute components along which the variation of attribute values is largest. For the benchmark datasets, the two principal components along which the attribute space shows the largest variation were identified and points in attribute space projected onto them. A first estimate of the number of clusters in attribute space can be obtained based on visual inspection of this projection. Clustering methods may also perform better on the transformed data than raw data even if the number of dimensions is not reduced. The Euclidean distances between points in attribute space were clustered using *k*-means after projecting each point along the principal directions.

The optimal number of clusters is evaluated with the implementation of the Calinski-Harabasz (CH) method [44] in MATLAB and was called through the evalcluster function. The number of clusters is varied between one and 10. For each selected number of clusters, the CH coefficient is calculated. The optimal number of clusters (*q*) is selected as the number of clusters for which the variation of the CH clusters showed an elbow. If the optimal number is 8 or 9, the maximum number of clusters was increased to 15 in order to verify if an alternative solution existed beyond 10 clusters.

Since *k*-means clustering can result in a partition that is a local minimum, a total of 100 replicas of clustering were performed on each data set in order to improve the sampling for finding an optimal clustering. It was found that *q* varied between 2 and 9 for all datasets as a result of diversity of pair attribute variation with superfamilies, even if visual inspection broadly identified 2 or 3 clusters. In order to achieve a consensus between k-means and visual inspection based number of clusters, a coarse-graining procedure is applied. For each of the *q* clusters, the mean value of each component of the attribute vector was calculated. The resulting mean attribute vectors for all clusters were clustered into two groups. The group with the largest mean attribute vectors was selected as representative of Region 1 for community detection. Since k-means clustering is unaware of any Separatrix that may demarcate Region 1 from the remaining data, some clusters that may belong to Region 1 are excluded if the they are closer to clusters from the intermediate region. Such errors are corrected by using the benchmark datasets and identifying the smallest mean attribute cutoff vector, *A*_E,m_, above which most attribute vectors in the cluster contribute to Region 1.

Another potential shortcoming of the *k*-means clustering method is that some data points which are well separated from a cluster may be incorrectly assigned to the cluster if the distance of these points from the cluster is smaller than the distance from any other cluster. In such cases, the incorrectly assigned points are outliers that should be excluded. In order to identify outliers, the value of *a*_l_ for all links contributing to Region 1 are collected and sorted in order of decreasing magnitude. Based on an implementation of a modified boxplot, the range of *a*_l_ values that correspond to outliers is identified. All links with outlier values of *a*_l_ are excluded and the remaining links are sorted based on the value of *d*. The resultant set of links, free from outliers, is then used for community detection. Partition density minimization based on the resulting set of links representative of Region 1 should then lead to a reasonable first approximation for the number of communities. As there is no need for any user input during this stage of data analysis, the outlined procedure for community detection is entirely unsupervised. Inspection of data at each stage is nonetheless prudent.

Since the scaled attribute vectors are four-dimensional objects for any sequence dataset, it would be beneficial to combine them into a composite variable whose variation reflects the collective variation of the attribute vectors. The distance *d* from a perfect alignments attribute vector is selected as such a composite variable. The community structure refinement procedure is applied to the community structure obtained by Partition density maximization. For this purpose, a simple grid in the (*s*_f_, *Z*_0_) plane in the range 1.01≤s_f_≤1.10 and 1.100≤Z_0_≤1.110 and grid point separation of Δs_f_ = 0.01 and ΔZ_0_ = 0.001 is constructed. Partition density maximization over the selected set of grid points is then used to identify the final refined community structure. To ensure that sufficiently large inter-community d_min_ values are considered, a final value of Z_m_>1 is enforced and this excludes some grid points from consideration. The community structure refinement procedure is entirely unsupervised, making the entire implementation of ACDC an unsupervised method.

#### 2.5.3. Comparison of predicted and ground-truth community structures

In order to quantitatively evaluate the performance of the ACDC method, the predicted community structure should be compared with the “ground truth” communities in the datasets. One measure of performance is simply comparing the number of communities. However, this does not provide any information on the similarity of predicted and “ground truth” communities. A number of measures of cluster similarity are available which compare the internal or external properties of clusters. Here, we utilize the *F*-measure [45, 46], which is a composite index based on the Precision and Recall of the communities.

Let *N* be the number of sequences, *n*_i_ the number of sequences in community *i* within the “ground truth” network, *m*_j_ the number of sequences in community *j* based on the selected method, and let *O*_ij_ be the number of sequences common to ground truth community *i* and identified community *j*. The precision (*P*) of cluster *j* is a measure of the fraction of sequences in the “ground truth” community that is correctly detected by the method and is evaluated as *P*_ij_ = *O*_ij_/*n*_i_. Similarly, recall (*R*) is defined as the fraction of sequences common to the detected and “ground truth” communities that is present in community *j*, and is defined as *R*_ij_ = *O*_ij_/*m*_j_. The *F*-measure then combines Precision and Recall into a single measure defined as

$$F=\frac{1}{N}\Sigma_{i}n_{i}\left[max_{j}\left\{\frac{2}{\frac{1}{R_{ij}}+\frac{1}{P_{ij}}}\right\}\right]$$ (2)

For a perfect community detection method, *P* = *R* = 1 and the corresponding *F*-measure is also 1. In the other extreme, in the case of complete failure when every community has exactly one sequence, both Precision and Recall are small, but not zero, and the corresponding F-measure is also small, but not zero. Note that a low F-measure does not necessarily imply bad performance, rather it indicates a harder task [47]. Here, the F-measure was calculated for the detected communities structure and compared to the “ground truth” communities for each sequence dataset.

#### 2.5.4 Outlier detection

A computationally simple method for outlier detection from a univariate sampling of data is the boxplot. In its simplest form this requires the calculation of the sample median(m), the first (q_1_) and third (q_3_) quartiles. As proposed by Tukey [48], for the set of observations X = {x_1_, x_2_, …,x_n_}, where all data is sorted in increasing order of value, data points outside the range [q_1_-1.5(q_3_-q_1_), q_1_+1.5(q_3_-q_1_)] are selected as outliers. For data sampled from a normal distribution, this range corresponds to a probability of 0.007 that a randomly selected data point will be outside the selected range. The resulting outliers roughly correspond to 0.7% of the data. However, this method performs well only for distributions that are mostly symmetrical within the inter-quartile region. There is no *a priori* reason to make such an assumption for sequence similarity data. Instead, the medcouple (mc)-based outlier detection method proposed by Dovoedo [49] is selected. The medcouple is a measure of skewness of the data[50] and is defined as

$$mc=\begin{array}{c} med\\ x_{i}\leq q_{2}\leq x_{j} \end{array}h\left(x_{i},x_{j}\right)$$ (3)

where h(x_i_,x_j_) is a kernel function and med corresponds to the median value of *h*(*x*_i_,*x*_j_). For *x*_i_≠*x*_j_,

$$h\left(x_{i},x_{j}\right)=\frac{\left(x_{j}-q_{2}\right)-\left(q_{2}-x_{i}\right)}{\left(x_{j}-x_{i}\right)}$$ (4)

For *x*_i_ = *x*_j_ = *q*_2_, let there be *k* observations *m*_1_ to *m*_k_ that are equal to the sample median, then

$$h\left(x_{j}-x_{i}\right)=\{\begin{array}{ll} -1 & i+j-1<k\\ 0 & i+j-1=k\\ 1 & i+j-1>k \end{array}$$ (5)

For right-skewed sample distributions, mc >0, for left-skewed distributions, mc<0, and for symmetric distributions, mc = 0. Building on the medcouple based outlier detection method of Hubert *et*. *al*[51], Dovoedo [49] proposed that points outside the range [*q*_2_-4e^(-1.93 mc)^(*q*_2_-*q*_1_): *q*_2_+4e^(2.18 mc)^(*q*_3_-*q*_2_)] should be considered to be outliers. The probability that a randomly selected data point lies outside the fences is maintained at 0.007. The stability of this proposed outlier detection method for skewed distributions and its simple implementation makes this method suitable for automated outlier detection.

## 3. Results

### 3.1. Attribute vectors reveal systematic variation of alignment attributes

**Fig 1:**
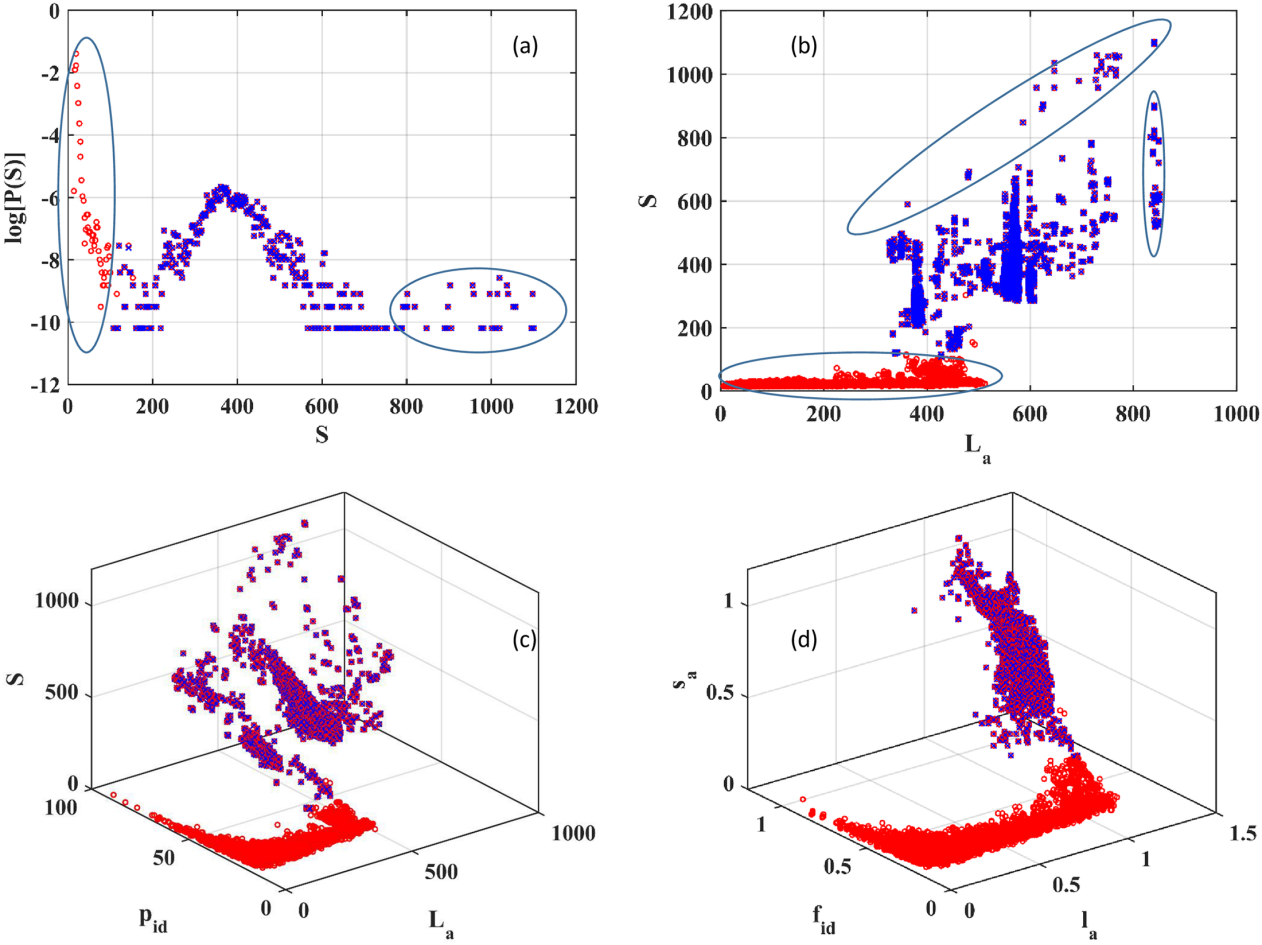
Alignment attributes from the Amidohydrolase superfamily can be represented in several ways. (a) Frequency distribution of scores (S), (b) Scatter plot of attribute vectors in the (L_a_, S) plane, (c) variation of attribute vectors in (L_a_, p_id_, S) space, and (d) the scaled attribute vectors in (l_a_, f_id_, s_a_) space.

A key difference between other SSN analysis methods and ACDC is the use in the latter of a new scaled attribute vector. The question thus arises as to whether the introduction of this new attribute improves the classification of pair alignments? A good selection of alignment attributes should (a) have enough variables to distinguish between different pair alignments, (b) show features in their distributions that allow for clear separation of the attribute vectors into regions that could be interpreted as pair alignments between homologous or unrelated sequence pairs, and (c) preferably reveal systematic variation in the data. To address these issues, the frequency distribution P(*S*) of alignment scores, scatter plots of the unscaled pair alignment attributes in the (*L*_a_, *S*) plane and in (*L*_a_, *P*_id_, *S*) space (here *P*_id_ is the percentage of identical residues), as well as the scaled pair alignment attributes in (*l*_a_, *f*_id_, *s*_a_) space were calculated. For all these choices of attributes, data based on intra-community links was also included in order to understand the ability of the selected attributes to distinguish between intra- and inter-community links. Results for the AH superfamily are presented in Fig 1 while data for all other superfamilies is presented in S1 Fig.

The frequency distribution of alignment scores is one of the simplest summaries of alignment information and is shown in Fig 1a for the AH superfamily. The distribution is peaked at small values of the alignment score, *S*, and shows at least three sub-populations. Since two alignments with the same S may be indistinguishable, it is not clear how insightful P(*S*) might be. Further, the small *S* region (at ~ 0<*S*<100) and the intermediate peak (at 100<*S*<700) overlap and separating the distributions into distinct domains is not straightforward. Another consequence of this overlap is that the intra-community alignment scores cannot be separated from the inter-community alignment scores.

To remedy this situation, increasing the dimensionality of the attribute data is one option. As an example, a projection of attribute vectors onto the (*L*_a_, *S*) plane is presented in 1(b). Now, there is improvement in separation of data into distinct regions with respect to the distribution of scores. Thus, the selection of these two attributes helps distinguish intra- from inter-community pair sequence alignments. At least four clusters of points can be discerned, shown in Fig 1b. There is no systematic variation between the clusters. Note that only the cluster with the smallest S values contains most of the inter-community attributes.

By going to three dimensions, a scatter plot of attributes in the (*L*_a_, *P*_id_, *S*) space leads to a more complicated distribution of points, shown in Fig 1c. Now, a large number of clusters is visible although no systematic overall trends are apparent, except for the increase in *L*_a_ at almost constant *S* for small values of *L*_a_, a feature present in Fig 1b but not Fig 1a. A second feature that is apparent at small *S* and *L*_a_ is the increase in *P*_id_ which is weakly dependent on *S* and *L*_a_. This feature cannot be discerned in Fig 1b. The introduction of scaled attributes transforms Fig 1c into Fig 1d where, instead of distinct clusters, there is a clear continuous variation of alignment attributes in attribute space. The separation of intra- from inter-community attributes is no worse than the unscaled attributes in three dimensions. The same overall improvement when using scaled attributes is apparent for all other gold standard datasets (see S1 Fig). Thus, attribute scaling brings out the hidden systematic variation of alignment attributes within a superfamily which would be missed with any of the other selections of attribute vectors considered here.

### 3.2. Overall distribution of points in attribute space is superfamily independent

In order for the variation of points in attribute space, as in Fig 1d, to be useful for partitioning the SSN, it is important to establish the generality of the variation of pair alignments in attribute space. Since the scatter plots reveal a large variation, it would be useful to coarse-grain attribute space in order to identify a less noisy trend. A local coarse-grained representation of the populated attribute space can be obtained by averaging the alignment attributes for neighboring points. To do so, a set of neighboring points has to be found. As it turns out, k-means clustering works by collecting points into clusters such that points within the cluster are closer to each other than to points in different clusters. With an appropriate selection of the number of clusters, a small number of points that are representative of the local features of the attribute space can be identified. The number of points in each cluster was selected to be approximately 100, except for the R15 dataset (see Methods-Data Sets) where all points are included. This represents a choice that is expected to be small enough that the averages represent local features and large enough that the averages are well behaved. The average attribute vector for each cluster was calculated and cluster indices ordered such that the scaled scores are in ascending order for the clusters.

**Fig 2:**
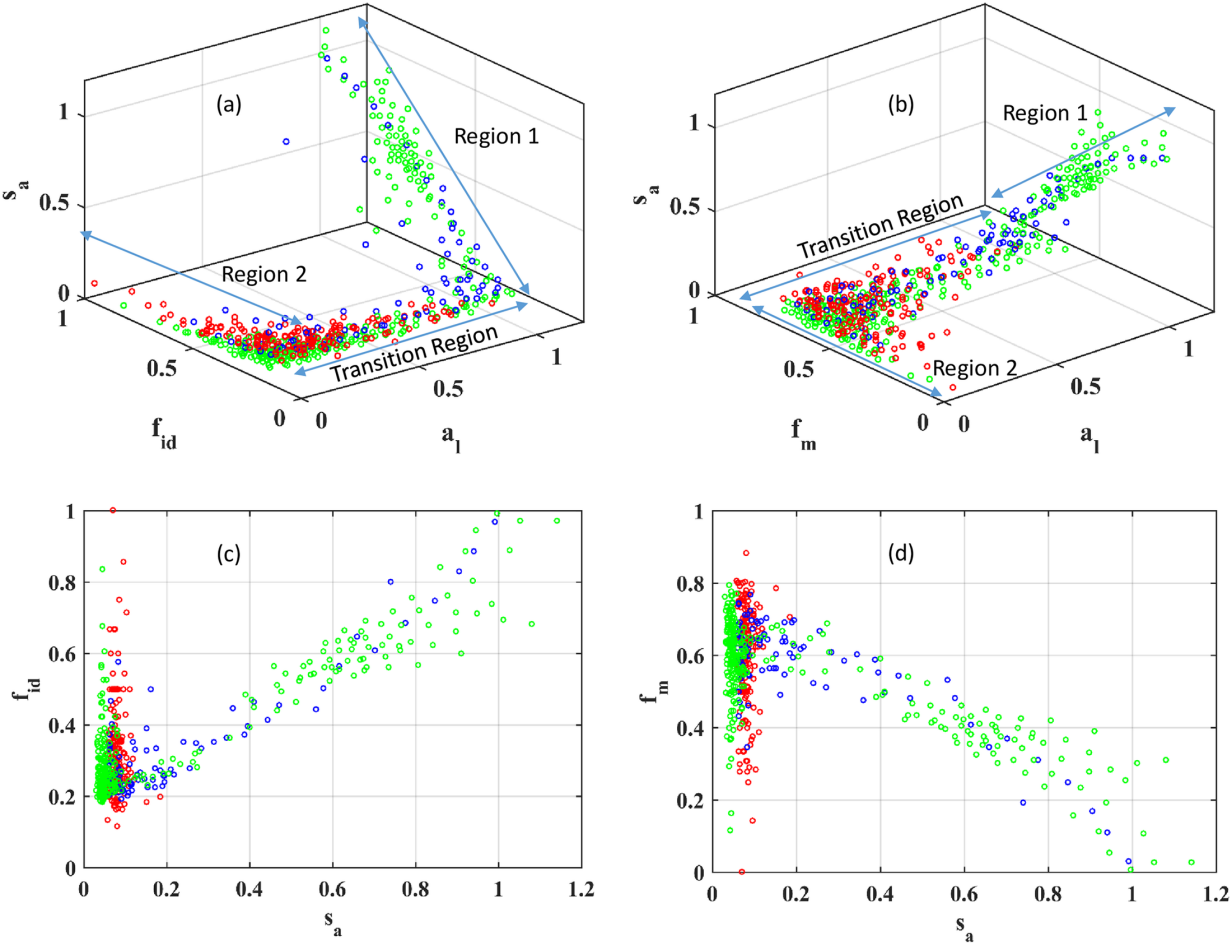
Distribution of attribute vectors in attribute space partition is superfamily independent. (a) in (*l*_a_, *f*_id_, *s*_a_) space, (b) in (*l*_a_, *f*_m_, *s*_a_) space, (c) in (*f*_id_, *s*_a_) plane, and (d) in (*f*_m_, *s*_a_) plane for the R15 dataset (in red), Amidohydrolase superfamily (in green), and Vicinyl Oxygen Chelatase superfamily (in blue).

In Fig 2, the variation of the coarse-grained attribute vectors is presented for the R15, VOC (Vicinyl Oxygen Chelatase), and AH datasets. The variation of points in (*l*_a_, *f*_id_, *s*_a_) and (*l*_a_, *f*_m_, *s*_a_) space clearly shows the presence of two distinct limiting regions, labeled Region 1 and Region 2 in the figure. Region 1 contains attribute vectors that could be thought of as starting from the highest attribute values (≈1) in Fig 2a, except for *f*_m_ which starts from its lowest values (≈0) in Fig 2b. As *s*_a_ decreases (rightmost points in Fig 2a and 2b), *l*_a_ values tend to stay mostly unchanged until *s*_a_ reaches a value smaller than ≈0.2 in Region 1. Given that *l*_a_ ≈1 in Region 1, such attribute vectors correspond to pair alignments that roughly cover the entire length of the shorter sequence in the pair alignment, akin to a global alignment. As the number of mutations increases over the entire length of the sequence, the alignment score, and therefore *s*_a_, decreases, while *f*_id_ decreases to about 0.3 and *f*_m_ increases to about 0.6. Thus, alignments in Region 1 correspond to sequence pairs that have not diverged significantly and are likely to be homologous.

For *s*_a_ smaller than 0.2, *l*_a_ starts to decrease from a value of about 1 in Region 1 to much smaller values in the intermediate region. The decrease in *l*_a_ implies that sequence pairs can be aligned only over small fractions of their sequence lengths. Along with this variation in *l*_a_, *f*_id_ gradually decreases from 0.3 to 0.2 (see Fig 2c) and *f*_m_ gradually increases from ≈0.6 to ≈0.8 (see Fig 2d) between Regions 1 and 2. Thus, the number of mismatched residues increases over the transition region. Together, these observations suggest that alignments in the intermediate region, going from Region 1 to Region 2, correspond to increasingly divergent pairs of sequences and may represent inter-community pair alignments or perhaps alignments between highly divergent sequence pairs within the same community.

In Region 2, *s*_a_ and *l*_a_ stay small, *f*_id_ starts increasing and *f*_m_ starts decreasing from its typical values in the intermediate region. The large values of percentage identity and small alignment lengths seen for Region 2 have previously been demonstrated to exist for non-homologous sequence pairs, for example, by Rost[35]. Thus, *f*_id_ = 0.3 or a percentage identity of 30% between two sequences need not correspond to homologous sequence pairs if the attribute vector lies in Region 2. For the R15 dataset, pair alignments primarily occupy Region 2 and part of the intermediate region. Since R15 contains sequences with very low sequence similarity, it is reasonable to propose that Region 2 contains pair alignments between unrelated sequences or very distantly related sequence pairs.

Attribute vectors for the AH and VOC datasets mostly follow the overall variation outlined in the preceding paragraphs although there is some superfamily dependent variation. It is anticipated that for other protein superfamilies, additional parts of attribute space may be available and the distribution of points in attribute space may differ from the pathway presented here. Such deviations may be indicative of a different evolutionary history for sequences in the superfamily.

The overall presence of two regions of different variation of attributes and an intermediate region between them in Fig 2a and 2b suggests a gradual transition in topology of attribute space between regions corresponding to significant and insignificant sequence similarity. Thus, coarse-graining of attribute vectors clearly demonstrates a transition path in attribute space and the changes in attributes along the pathway. To the best of the author’s knowledge, the overall variation in attribute space has never been presented before in any community detection method for any sequence dataset.

### 3.3 Detecting communities with ACDC

**Fig 3:**
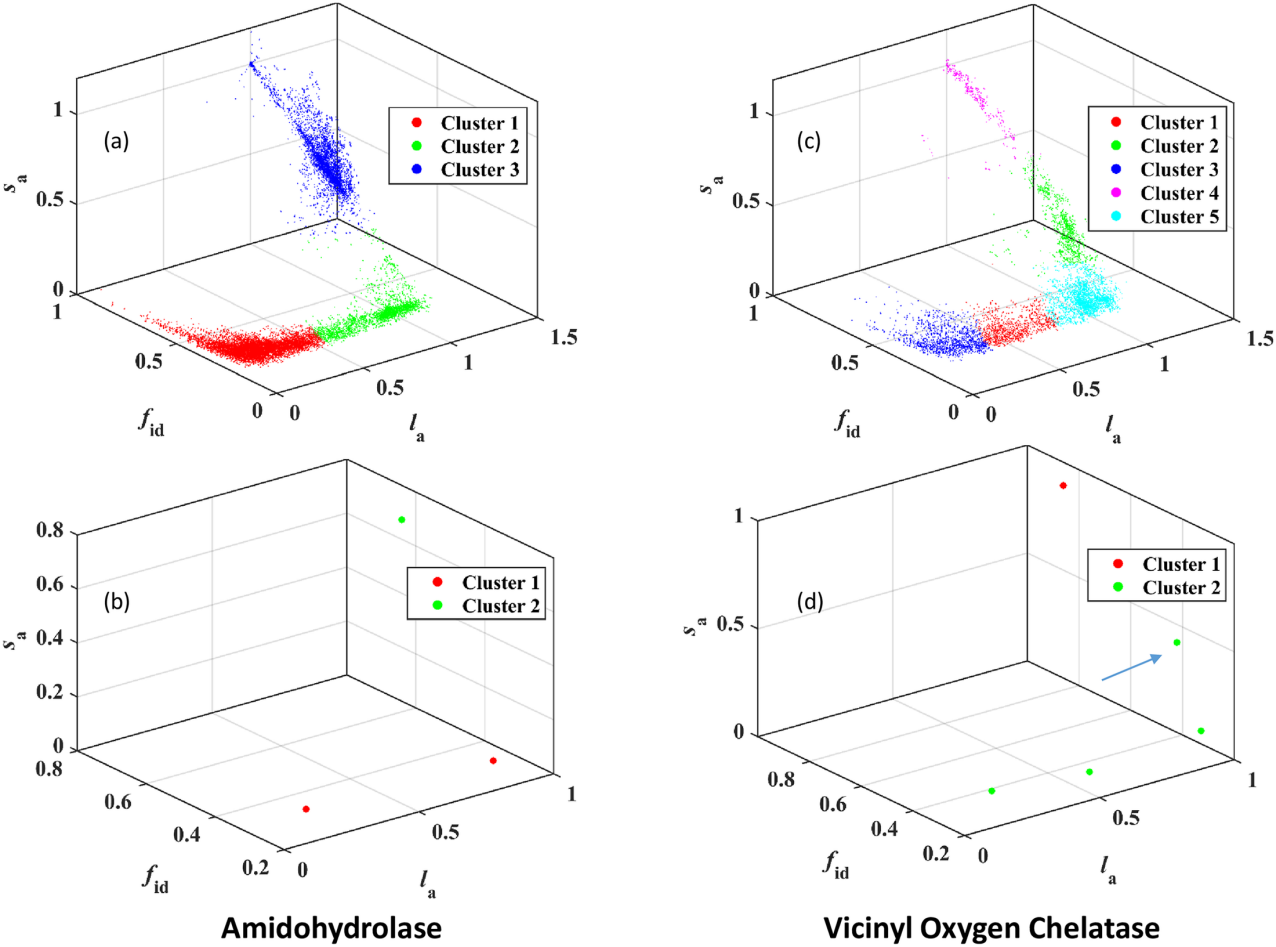
Partition of attribute vectors and mean attribute vectors into clusters by k-means clustering. (a) Cluster assignment for each attribute vector from the Amidohydrolase superfamily, (b) Assignment of each clusters mean attribute vector for the Amidohydrolase superfamily to Region 1 or otherwise, (c) Cluster assignment for each attribute vectors from the Vicinyl Oxygen Chelatase superfamily, and (d) Assignment of cluster mean attribute vector for the Vicinyl Oxygen Chelatase superfamily. Clusters with highest *s*_a_ values exceeding 0.3 are included in Region 1 for both superfamiles. An error in cluster assignment to Region 1 is indicated in (d) with an arrow and is corrected in the analysis.

Having identified a scaled attribute vector representing pair alignments, the overall distribution of points in attribute space and the potential implications of different regions in attribute space, now the structure of attribute space is used to detect communities. In ACDC, the first step is to cluster all attribute vectors in the 4-dimensional space. The optimal number of clusters was identified using the variation of the Calinski-Harabasz coefficient (S2 Fig) and the distribution of points in each cluster is presented for the AH and VOC superfamilies in Fig 3a and 3b respectively. The differences in the distribution of points in attribute space leads to different numbers of clusters. The number of clusters contributing to Region 1 for the AH and VOC superfamiles are 1, Cluster 3 in Fig 3a, and 3, Clusters 1 and 3 in Fig 3b, respectively. There is no spatial demarcation of these clusters from the adjoining cluster, cluster 1 for AH and cluster 2 for VOC. Such a variation is expected given the continuity of attribute values.

The second step of ACDC involves identifying the mean attribute value for each cluster followed by clustering these mean attribute vectors. The mean cluster values are presented in Fig 3c and 3d for the AH and VOC superfamilies respectively. For the AH superfamily, there is clear separation between the large mean *s*_a_ valued clusters and the other two clusters. In the case of VOC, clustering of the mean attribute vectors leads to two clusters as shown in Fig 1d. One cluster, Cluster 1, in Fig 3d, is a natural constituent of Region 1 and contains the largest *s*_a_ attribute vectors. Of the remaining attribute clusters, one (indicated with an arrow in Fig 3d), has a large value of the mean *s*_a_, but is included in the second cluster of mean attribute vectors. As discussed in the Methods section, imposing a lower cutoff on the mean attribute vector can lead to better assignment. For VOC, a cutoff of *s*_a_ = 0.41 correctly assigns this erroneously assigned cluster to Region 1. However, to make it more generally applicable, the minimum mean attribute vector *A*_E,m_, was selected such that it satisfies the condition *s*_a_ ≥0.30. The value of 0.30 is selected since (a) most attribute vectors in the intermediate region have *s*_a_>0.24 and (b) the change in variation from Region 1 to the intermediate region starts at *s*_a_≈0.30.

Following the selection of attribute space clusters for Region 1, the *l*_a_ values for all selected attribute vectors are collected and outlier analysis performed in order to finalize the set of attribute vectors to be analyzed in the next stage. As clear in both Fig 3a and 3b, the distribution of points in Region 1 is broad and has disconnected regions that clearly contain outliers. Links corresponding to these outliers are removed (see Methods) and the shortlisted attribute vectors are considered for the optimization of D. Analysis of these shortlisted attribute vectors in 4-dimensional space is simplified by introducing the collective variable, *d*, which measures the distance of each attribute vector from a perfect alignments attribute vector. The list of attribute vectors is sorted in increasing order of *d*. For each cut-off value of *d*, all links with smaller *d* values are collected, the community structure identified and the magnitude of *D* evaluated.

**Fig 4:**
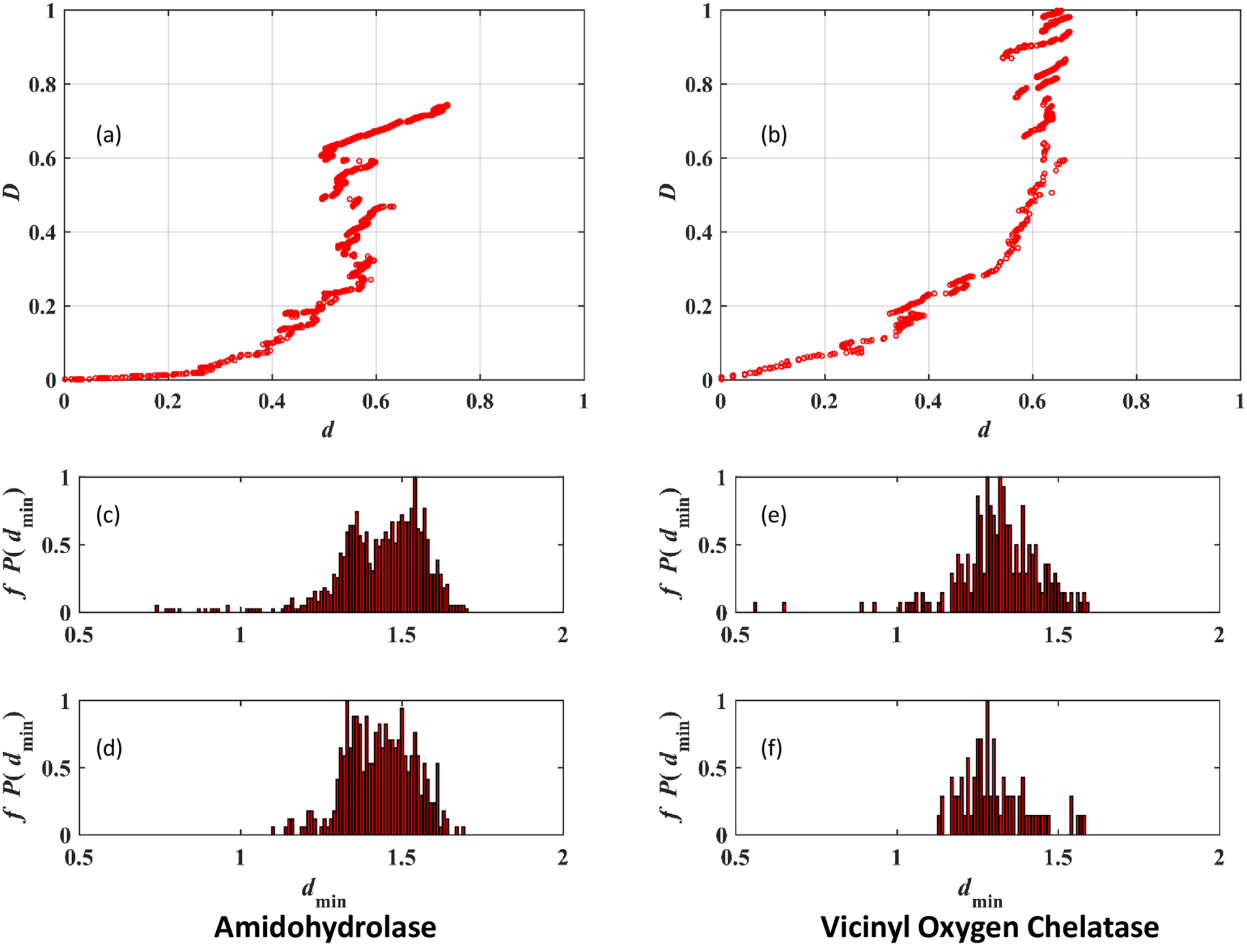
Community structure optimization and refinement with ACDC. (a) Optimization of partition density (*D*) with distance *d* from the perfect alignments attribute vector for the Amidohydrolase superfamily, (b) Partition density (D) versus distance *d* for the Vicinyl Oxygen Chelatase superfamily, (c) the distribution of inter-community *d*_min_ before community refinement and (d) after community refinement for the Amidohydrolase, (e) the distribution of inter-community d_min_ before community refinement, and (f) after community refinement for the Vicinyl Oxygen Chelatase superfamily. The distributions have been scaled such that the bin with the largest frequency has a value of 1.

The variation of *D* as a function of *d* is presented in Fig 4a and 4b for the AH and VOC superfamilies. For both superfamilies, *D*(d) shows a non-monotonic variation with d with large fluctuations occurring associated with small changes in *d*. Links with similar values of *d* when added to different communities may change the shape of these communities, possibly by merger of communities, thereby leading to these large fluctuations. Since the objective is to optimize community structure, the variation with *d* is not as important as the community structure obtained by maximization of D.

Defining *d*_min_ to be the minimum inter-community value of *d* for a pair of communities, the last step of community refinement in ACDC is based on the distribution of *d* and includes contributions from all pairs of communities present in the network. After the optimization of *D*, the distribution of inter-community *d*_min_ values is calculated and the results are presented in Fig 4c and 4e for the AH and VOC superfamilies, respectively. Both figures contain a broad distribution centered at *d*_min_≈1.5 and contains a small number of points at small values. If the broad distribution were to be interpreted as arising due to some unspecified evolutionary process, the small *d*_min_ components would be thought of as outliers with respect to the distribution. These outliers are selected (see Methods), and the corresponding communities are merged iteratively. After the merger of communities, the resulting distribution of *d*_min_ values is presented in Fig 4d and 4f. As expected, the low *d*_min_ outliers are eliminated. The broad distribution on the other hand becomes less noisy and can be approximated, to first order, as a Gaussian distribution. A few large *d*_min_ value outliers are apparent, particularly for the VOC superfamily, and they perhaps represent the most divergent pairs of communities within the superfamilies.

**Fig 5:**
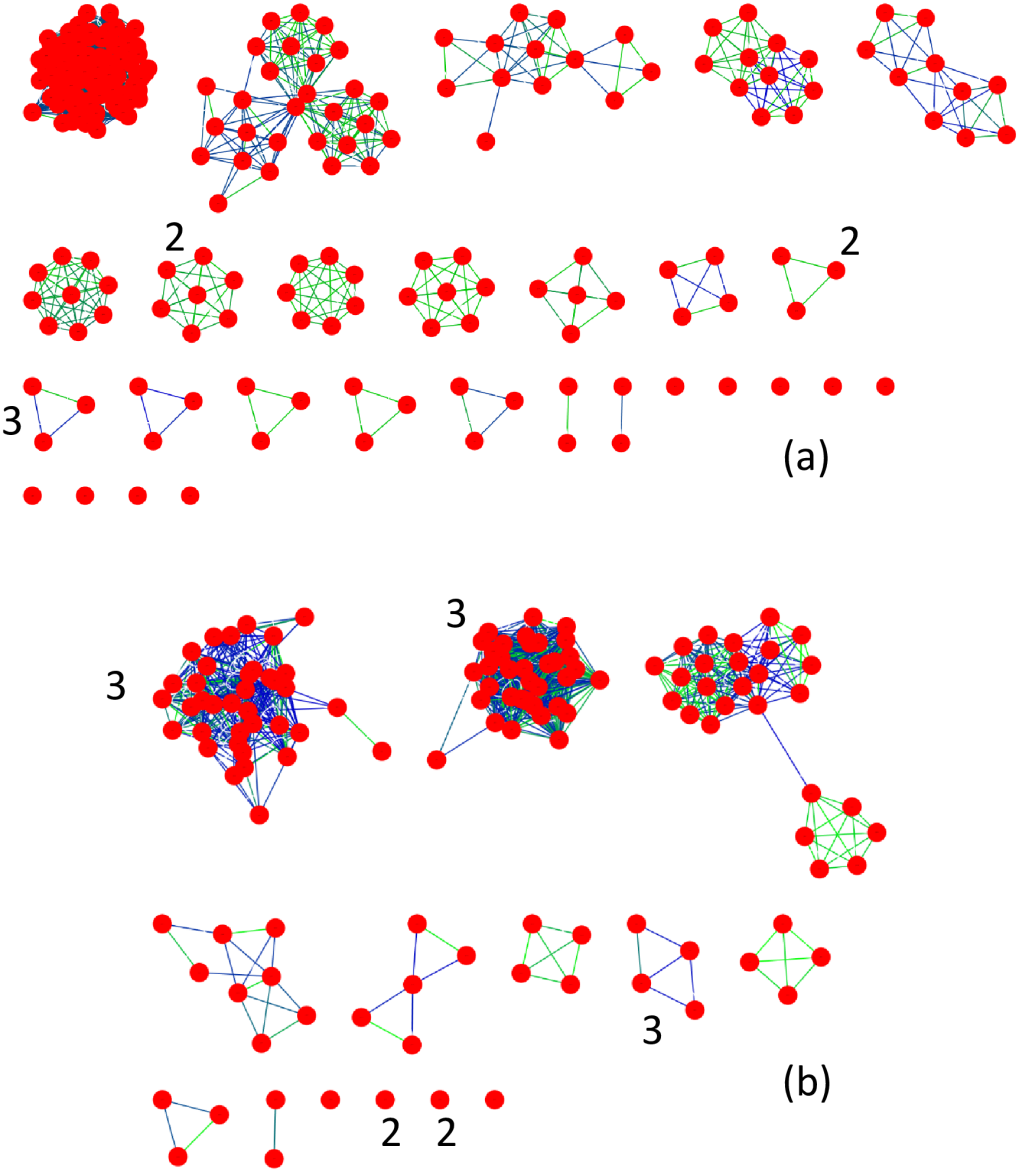
Community structure based on ACDC and the resulting error types. (a) Amidohydrolase superfamily community structure and (b) Vicinyl Oxygen Chelatase superfamily community structure. Each node (red) corresponds to a sequence. The set of links obtained with the ACDC method are also shown in increasing order of d from 0 (green) to the maximum d value (blue). Also, shown are the type of errors associated with communities (if any). Communities with Type 2 errors are merged in the “ground truth” community structure. Type 3 communities are formed by merger of independent “ground truth” communities.

The community structures for the AH and VOC SSNs predicted by ACDC are presented in Fig 5. In the figure, each node is connected by a link whose color is an indicator of the value of *d* for that link. Maximization of D invariably leads to highly connected communities, and this is indeed the case. Some communities have irregular shapes but these are likely a consequence of the pair similarity. An example of such a community is shown in Fig 5b (top row rightmost) where the community appears to be formed by merger of two communities; however, these correspond to isofunctional sequences from the same community.

Next, the community structures predicted by ACDC are compared with the ground truth community structures based on the number of communities and the F-measure. The results for all datasets are summarized in Table 2 and the intermediate data are presented in S2 Fig. The F-measure values for most datasets are around 0.9 or higher, which is indicative of good community detection. ACDC predicts a smaller number of communities for the Amidohydrolase, Vicinyl Oxygen Chelatase, and Gold Standard datasets, which is likely a consequence of merger of communities that ought to be distinct. The SSN for the Crotonase, Enolase and Haloacid Dehalogenase superfamilies are over-fragmented, perhaps due to the presence of fragmented communities. The worst performance of ACDC is for the VOC superfamily for which the F-measure≈0.78 and this is sub-optimal.

**Table 2:** Performance evaluation of the ACDC and GridS methods.

	Ground Truth	ACDC		GridS	
Dataset	#Communities	#Communities	F-measure	#Communities	F-measure
**Amidohydrolase**	29	28	0.9859	29	0.9877
**Crotonase**	16	16	0.8474	14	0.9741
**Enolase**	9	10	0.9684	9	0.9797
**Haloacid Dehalogenase**	20	23	0.9825	20	1
**Vicinyl Oxygen Chelatase**	17	14	0.7821	17	0.8315
**Gold Standard**	91	86	0.9368	86	0.9484

To explore the reasons for the imperfect agreement of the ACDC method, the community membership of sequences from the AH superfamily is analyzed. One community based on the ACDC method contains three sequences, gi|4033703 (NCBI Acc # Q52725.2), gi|6226558 (NCBI Acc #: P72156.2), and gi|11890745 (NCBI Acc #: AAG41202), which are assigned different functions in the gold standard database, namely, s-triazine hydrolase, atrazine chlorohydrolase, and melamine deaminase, respectively. For pair alignments between these three sequences, S = (145.8, 152.5,301.1) for the pairs (gi|4033703, gi|6226558), (gi|4033703,gi|11890745) and (gi|6226558, gi|11890745). These alignment scores are high and imply that they have significant sequence similarity. As a consequence of this similarity, these sequences constitute one community instead of the three expected based on the “ground truth” community structure. In Fig 5a, this Type 3 error is shown for one community. For the VOC dataset, additional occurrences of Type 3 errors are shown in Fig 5b. Note that within the limitation of a two-dimensional projection of the network and the visualization scheme employed here, the presence of overlapping community structure is not apparent. Manual inspection of the merged community nodes does not provide support for interpreting overlapping communities as the cause of Type 3 errors.

It would be tempting to infer that the sequences involved in Type 3 errors either correspond to multifunctional enzymes with as yet unexplored functional similarity or that mutations in the active site have significantly altered their substrate preference. Alternatively, the choice of the substitution matrix may somehow result in pair alignments which are incorrectly assigned a high score for these sequences. In any case, the potential presence of such sequences with high sequence similarity scores but with different functions makes it difficult to assign them to separate communities. Hence, caution must be exercised in judging community detection methods.

A second type of error that arises is where a “ground truth” community splits into two or more communities. By definition, a community contains nodes with more intra-community than inter-community similarity. It was verified that this condition is satisfied for the split communities found in both AH and VOC datasets. Such Type 2 errors are shown in Fig 5 for both superfamilies for which only two fragments result from the error. The fragmentation of a “ground truth” community is a consequence of the absence of a high-enough score for the pair alignment between sequences in each fragment. Such errors are likely to be a product of the alignment and do not necessarily reflect shortcomings of the community detection method.

A good test of any community detection method for SSNs is its ability to distinguish between communities that belong to distinct superfamilies when sequences from multiple superfamilies are present. The gold standard dataset is composed of five superfamilies and the number of “ground truth” communities in the Gold Standard dataset is 91. Assuming no overlap, the union of the set of communities identified by ACDC for each superfamily within the Gold Standard dataset leads to a total of 91 communities (from Table 2), in agreement with the “ground truth” number of communities. However, when all sequences are included together, the number of communities detected by ACDC is 86, instead of the “ground truth” number of 91. Either ACDC leads to Type 3 errors involving inter-superfamily community merger or from intra-superfamily community merger. Examination of superfamily membership of all nodes in each community did not reveal any instances of the mergering of communities from different superfamilies. Thus, ACDC erroneously merges communities from the same superfamily in the presence of additional superfamilies. Nevertheless, the high F-measure of 0.9368 indicates good overall performance of ACDC.

### 3.4 Performance comparison of ACDC with other community detection methods

In a recent evaluation of the performance of different clustering/community detection methods on SSNs [33], TransClust was found to be the most successful method with an F-measure≈0.914 for the Gold Standard dataset. At the outset, an F-measure value of 0.9368 for ACDC suggests that it outperforms TransClust, at least for the GS dataset. However, the SSN used with ACDC is based on the BLOSUM50 substitution matrix and differs from the SSN generated with the BLOSUM62 substitution matrix employed by Bernardes *et al* [33], complicates a direct comparison. Furthermore, it is important to note that ACDC utilizes a multidimensional pair similarity attribute vector, unlike TransClust which is typically applied to SSNs based on the choice of negative logarithm of pair similarity E-values as the alignment attribute. Unfortunately, apart from ACDC, no community detection methods based on multidimensional attribute vectors have been applied to SSNs. Therefore, a straightforward four-dimensional grid search (GridS) is employed for comparison with ACDC. GridS assumes the existence of a Separatrix cutoff attribute vector that partitions attribute space into a region that contains links relevant for community detection and the rest of attribute space. The detailed implementation of the GridS method is presented in Methods.

For comparison of GridS with ACDC, the maximum F-measure and the corresponding number of communities are summarized in Table 2. All F-measure values based on GridS are superior to ACDC for all datasets. For the Gold Standard dataset, the best F-measure value of 0.9484 exceeds the corresponding values of 0.9368 based on ACDC and 0.914 obtained with TransClust [33]. The best performance of GridS is for the Haloacid Dehalogenase superfamily for which the exact “ground truth” community structure is reproduced. The worst performance of GridS is for the VOC dataset, for which the F-measure of 0.8315 is only slightly better than the value of 0.7821 for ACDC. Thus, sequence similarities for the VOC dataset may be symptomatic of problems in using sequence similarity to distinguish between mechanistically distinct sequence communities or perhaps due to the choice of substitution matrices. Turning to the number of communities, GridS leads to the “ground truth” number of communities except for the Crotonase and Gold Standard datasets, for which the number is underestimated. Overall, GridS leads to the best community structure for the “ground truth” SSNs.

Given the optimal set of cutoff attribute vectors identified by GridS, the validity of the assumption that Region 1 contains the set of links, with large *l*_a_ values, that are most relevant to community structure detection can be tested. The set of cutoff attribute vectors, {(*l*_a,c_, *f*_id,c_, *f*_m,c,_ s_a,c_)}, that leads to the largest F-measure value for each dataset are summarized in Table 3 as a range of attribute values. The best solution does include the large *l*_a_ region of attribute space, as expected. However, attribute value combinations that extend to small values of *l*_a_ are also possible. These small *l*_a_ attribute vectors represent distant relationships which perhaps provide alternative routes for connecting nodes while keeping the F-measure unchanged. Thus, the GridS method supports the assumption that links with large *l*_a_ are most relevant to community structure detection.

**Table 3:** Cutoff attribute vector range based on the GridS method.

Dataset	Cutoff attribute vector range
**Amidohydrolase**	(0.95–0.96, 0.32–0.33, 0.52–0.83, 0.28)
**Crotonase**	(0.14–0.71, 0.38, 0.48–0.80, 0.14–0.33)
**Enolase**	(0.99, 0.31, 0.49–0.69, 0.25/0.28)
**Haloacid Dehalogenase**	(0.39–0.71, 0.31, 0.46–0.62, 0.29)
**Vicinyl Oxygen Chelatase**	(0.10–0.81, 0.38, 0.0–0.83, 0.14–0.28)
**Gold Standard**	(0.19–0.25, 0.37, 0.0–0.80, 0.24)

The large variation in *f*_m_ values for all datasets suggests that community structure is least sensitive to the fraction of mismatched residues. In contrast, *f*_id,c_ and *s*_a,c_ values for best community structure are typically in the range 0.31≤*f*_id,c_≤0.38 and 0.14≤*s*_a,c_≤0.33. The variation of the former attribute is consistent with the choice of 30–40% identity of residues for homologous sequences [8, 35]. The narrow range of values for *s*_a,c_ suggests that homology detection may benefit from including the scaled score as well.

**Fig 6:**
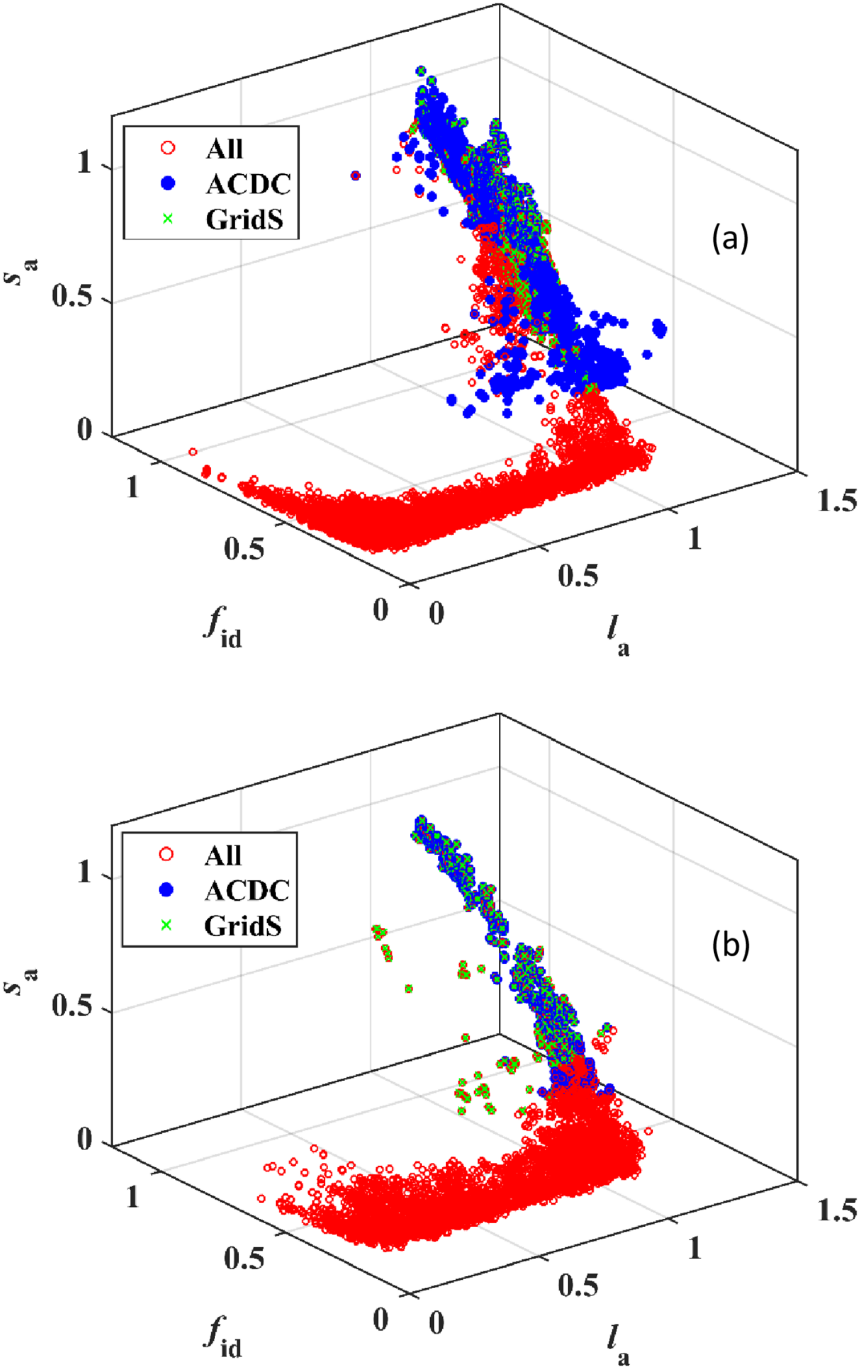
Distribution of links in attribute space relevant for community identification. (a) For the Amidohydrolase and (b) Vicnyl Oxygen Chelatase superfamily. All links (red), links selected by ACDC (blue), and links that give the maximum F-measure based on GridS (green) are presented for comparison. The cutoff attribute vectors selected by GridS for the Amidohydrolase and Vicnyl Oxygen Chelatase superfamiles are (0.95, 0.32, 0.83, 0.28) and (0.10, 0.38, 0.80, 0.22), respectively.

Given the larger F-measure values obtained with GridS, it would be useful to identify potential differences between the optimal GridS and ACDC solutions that may contribute to the better performance of former. During a grid search, each value of F-measure can be obtained for a number of different attribute cutoff vectors. For the AH and VOC datasets, the cutoff attribute vectors (0.95, 0.32, 0.83, 0.28) and (0.10, 0.38, 0.80, 0.22) are selected as they deviate most from Region 1. The attribute vectors that satisfy these cutoff values in GridS are shown in Fig 6 along with those predicted by ACDC and all available links in the SSN.

For both the AH and VOC datasets, ACDC and GridS excluded most of the available links and selected only the large *l*_a_ region of attribute space. For the AH dataset, ACDC selects more links at smaller *l*_a_ values than GridS even though the F-measure values are quite similar. Given that GridS leads to 29 communities instead of the 28 found by ACDC, it would appear that the additional links included in ACDC may lead to a Type 3 error that is avoided in GridS. For the VOC dataset, GridS selects a number of links at small *l*_a_ values that are not included by ACDC. It was verified that including these additional links to the set of links identified by ACDC does not change the number of communities. GridS excludes a few links at small *s*_a_ values that are included by ACDC. Given that GridS finds 17 communities instead of the 14 found by ACDC, it is likely that the links excluded by GridS, as compared to ACDC, lead to a larger number of communities. The missing links in GridS occupy the region near the inter-cluster boundary, as shown in Fig 3c, and excluding them in ACDC may not be straightforward. Thus, small differences in the set of selected links, probably in the inter-cluster region of ACDC, may be responsible for differences in ACDC and GridS.

## 4. Discussion

An SSN is a network where each node corresponds to a protein (or nucleotide) sequence and each link indicates a similarity relationship between pairs of sequences. As with any real or artificial network, an important mesoscopic structural motif in a network is its community structure. For protein SSNs, each community corresponds to a functionally (and evolutionarily) related set of sequences and groups of related communities constitute a superfamily. Thus, community structure of SSNs is an important tool for sequence classification. In this manuscript, a new method for detecting the community structure in protein SSNs is presented.

A crucial feature of an SSN is the similarity between pairs of sequences which can be quantified with a number of attributes such as the alignment score, fraction of identical residues, fraction of mismatched residues and the alignment length. These standard alignment attributes are automatically output by most sequence pair alignment programs. In order to differentiate between two pair alignments with the same alignment score, a novel sequence similarity attribute vector is constructed by supplementing the alignment score with standard alignment metrics, i.e., the alignment length, percentage identity and percentage of mismatched positions. Invoking a natural length and score scale for each pair alignment, a scaled attribute vector is proposed as a link attribute for the SSN. Now, each scaled attribute vector corresponds to a point in four-dimensional attribute space.

The distribution of points in attribute space varies continuously between two limiting regions via an intermediate region. To the best of the author’s knowledge, this attribute scaling and the resultant distribution of points in attribute space is absent from published literature. The two limiting regions were identified as a high sequence similarity Region 1 at large *l*_a_ values, and low sequence similarity Region 2 at small *l*_a_ values. For both these regions, there is substantial variation in the all alignment attributes, except *l*_a_. In Region 1, *f*_id_≥0.3 for all attribute vectors and they primarily correspond to intra-community sequence pairs which are expected to be evolutionarily related, *i*.*e*., they are homologous, and likely to be structurally similar [8, 35, 52].

In the intermediate region, 0.2≤*f*_id_≤0.3, sequence pairs belong to the twilight zone of sequence homology. About 10% of all such sequence pairs in the twilight zone have been estimated to share the same tertiary structure[35]. Since the intermediate region appears to contain a sizable fraction of inter-community attribute vectors (see Fig 1d and Figure d in S1 Fig), the percentage of such homologous sequence pairs in the intermediate region may differ from 10% and is superfamily dependent. It is likely that pair attribute vectors in this region correspond to divergent domains shared between distantly related sequence pairs.

Approaching Region 2, *l*_a_ decreases with respect to the intermediate region and *f*_id_<0.2 at first. Such *f*_id_ values correspond to the midnight region of sequence homology. The fraction of intra-community attribute vectors in this region is expected to be very small highlighting the problem of identifying homologous sequences in the midnight region. Such pair alignments may correspond to subdomain fragments with large similarity and perhaps represent “ancestral” peptides [53] shared by sequences in a superfamily. Alternatively, they may correspond to sequence fragments that share significant sequence similarity independent of evolutionary origin. Further analysis will be required to clearly identify the significance of this region.

The new community detection method, ACDC, makes use of the structure of the distribution of attribute vectors in attribute space. It is based on the hypothesis that links in Region 1 correspond to homologous sequence pairs that form the minimal set of links required for community detection. Such links in Region 1 are then identified as components of a subset of clusters identified with *k*-means clustering of points in attribute space. Based on a suitable choice of outliers, the minimal set of attribute vectors is shortlisted. Network structure is then utilized during partition density maximization for a first solution to the community structure. Community structure is then refined in order to eliminate Type 1 errors and minimize Type 2 errors which arise when the minimum inter-community similarity, *d*, is small than or slightly larger than the maximum intra-community similarity. The entire process is set up as an unsupervised method with no user specified parameters.

The performance of ACDC was evaluated by comparing the number of communities and the F-measure values with respect to “ground truth” community structure of the gold standard datasets of Brown et al[38] as well as the five functionally distinct protein superfamiles that constitute it. For the entire dataset, the F-measure value of 0.9368 based on ACDC exceeds a recently published best F-measure value of 0.914 obtained with TransClust [33]. At least for the Brown dataset[38], ACDC is the best performing community detection method for SSNs. For most constituent superfamilies, ACDC leads to F-measure values of about 0.9 or higher, except for the Vicinyl Oxygen Chelatase superfamily for which the F-measure of 0.78 suggests poor reproduction of the “ground truth” community structure. Analysis of the errors in community detection reveals (a) the partition of communities due to smaller similarity between sequences belonging to the two fragment communities or (b) merger of communities due to higher similarity between sequences that belong to different communities. Such errors may arise due to shortcomings of the substitution matrix used to calculate sequence similarity attributes. A study of the effect of the substitution matrix on community detection in SSNs using ACDC and its application to sequence databases with more distantly related homologous sequence will be presented in a following manuscript.

In order to compare the performance of ACDC to a method that is based on multidimensional attribute vectors, a four-dimensional grid search GridS was performed. The set of cutoff attribute vectors that lead to the best F-measure for all datasets was identified. GridS leads to F-measure values that exceed ACDC for all datasets. For the Gold Standard dataset, the GridS F-measure of 0.948 is much better than ACDC or TransClust. Note that this F-measure value is not significantly smaller than the F-measure of 0.959 that Bernardes *et al*. [33] obtained for the same dataset using a Hidden Markov Model profile-profile similarity network. However, note that a grid search by itself cannot be used to predict community structure, unless coupled with some criterion for selecting a best prediction in some efficient manner. It is hoped that future developments in community detection will benefit from comparison of their performance with benchmark GridS results. Comparison of the set of attribute vectors selected by ACDC and GridS indicates that ACDC includes links that contribute to the inter-cluster region that are excluded by GridS. For ACDC to perform better, this boundary region will have to be addressed in subsequent work.

ACDC incorporates partition density optimization, which was originally formulated in the context of link community detection; link communities in turn lead to the identification of overlapping communities of nodes. Since overlapping communities may arise in SSNs of multi-domain protein sequences the question arises as to whether ACDC can detect them. Among the superfamilies considered here for validating ACDC, the Enolase superfamily contains multi-domain sequences, each carrying the following PFAM domains pairs or one domain from each pair: (PF02746 and PF01188) or (PF03592 and PF00113) or (PF05034 and PF07476). The Enolase superfamily contains 9 “ground truth” communities and ACDC detects 10 communities. At least, then, for this example of a multi-domain sequence superfamily, ACDC performs well and the F-measure for the resulting community structure is 0.9684. Due to alignment length scaling and the preferential selection of attribute vectors with large scaled aligned lengths, ACDC preferentially selects alignments over the full length of the shorter sequence. As a result, multi-domain sequences aligned over the entire length of the shorter sequence are likely to be selected by the method and these will minimize the presence of overlapping communities. It is certainly possible that ACDC may not be as successful for other more complicated multi-domain sequence superfamilies. Then, the use of other overlapping community detection methods may be necessary. Further investigation is required to address this issue.

A general shortcoming of link based community detection methods is their scalability. For a superfamily with N sequences, there are n = N(N-1)/2 links in the SSN. For large superfamilies, such as the alpha/beta hydrolase fold super-family, with N≈O(10^5^) sequences, the number of links n≈O(10^10^) is clearly a huge number. A first step towards improving the performance of any SSN analysis method on large sequence datasets is to filter the dataset such that no two sequences have a percentage identity exceeding 99% or 95% identity is included. There are two computationally demanding steps in ACDC; k-means clustering and partition density optimization. Efficient implementations of k-means-like or grid based clustering methods have a time-complexity of O(nk) or O(n), where k is the number of clusters, or grid size [54]. In the partition density optimization step, all links are sorted by the distance d, a process that has a time complexity of O(n log n). From the sorted list of attributes, links are added to the network in increasing magnitude of d until a maximum value of the partition density is reached. Graph traversal and checking if two nodes are connected have time-complexities of O(n+N) and O(1)[55]. So, partition density optimization should scale reasonably well. As these two computationally demanding steps have reasonable scaling behavior, ACDC can be used for community detection in large sequence datasets, particularly once parallel implementations have been incorporated.

Most methods for community detection utilize one of, for example, the negative logarithm of the E-value, score, percentage identity, as link attributes. For each choice of attribute, a different SSN is obtained although the community structure is hopefully similar. The set of SSNs then comprises a multiplex network[56], one network for each attribute, and the community structure represents a balance between link attributes at each layer of the network. By integrating all attributes, ACDC in effect serves as a community detection method for multiplex networks. Although formulated for SSNs, it should be possible to extend ACDC to the analysis of network structure in multiplex networks. In any case, given the simplicity, intuitive interpretability, and unsupervised nature, it is hoped that the ACDC will prove useful for the scientific community.

## Supporting information

S1 Table Pair alignments with same scores can have different sets of alignment characteristics. (PDF)

S2 Table Mapping of Gold Standard dataset sequence gi numbers to NCBI accession numbers. (CSV)

S1 Fig Various representations for alignment attributes from the validation sequence datasets. (PDF)

S2 Fig Summary of results from all stages of ACDC for all sequence datasets. (PDF)

S1 Dataset All pair alignment data in BLAST tabular format for the Amidohydrolase superfamily. (TXT)

S2 Dataset All pair alignment data in BLAST tabular format for the Crotonase superfamily. (TXT)

S3 Dataset All pair alignment data in BLAST tabular format for the Enolase superfamily. (TXT)

S4 Dataset All pair alignment data in BLAST tabular format for the Haloacid Dehalogenase superfamily. (TXT)

S5 Dataset All pair alignment data in BLAST tabular format for the Vicinyl Oxygen Chelatase superfamily. (TXT)

S6 Dataset All pair alignment data in BLAST tabular format for the Gold Standard dataset. (TXT)

S7 Dataset All scaled attribute vectors for the Amidohydrolase superfamily. (TXT)

S8 Dataset All scaled attribute vectors for the Crotonase superfamily. (TXT)

S9 Dataset All scaled attribute vectors for the Enolase superfamily. (TXT)

S10 Dataset All scaled attribute vectors for the Haloacid Dehalogenase superfamily. (TXT)

S11 Dataset All scaled attribute vectors for the Vicinyl Oxygen Chelatase superfamily. (TXT)

S12 Dataset All scaled attribute vectors for the Gold Standard dataset. (TXT)
