# Supplementary material for: Community detection in sequence similarity networks based on attribute clustering
Source: PLoS One. 2017 Jul 24;12(7):e0178650. doi: 10.1371/journal.pone.0178650 (PMC5524321; doi:10.1371/journal.pone.0178650)
Supplement: S1 Table — (PDF) [file pone.0178650.s001.pdf]

Table S1. Pair alignments with same Scores can have different sets of alignment characteristics.

Sequence 1	Sequence 2	% Identify	Alignment Length	# Mismatches	#Gaps	E-val	Score
gi|16554592	gi|1706653	84.26	432	68	0	2.8E-142	500
gi|17367183	gi|50926833	91.18	431	38	0	2.9E-142	500
gi|13124248	gi|4416387	89.01	373	41	0	2.8E-127	450
gi|16975437	gi|1169532	84.4	436	62	6	3.2E-127	450
gi|16272483	gi|12313641	63.24	574	203	8	6.2E-112	400
gi|11999247	gi|6015090	83.47	363	60	0	2.9E-112	400
gi|886331	gi|14579320	53.73	577	259	8	7E-97	350
gi|4416379	gi|2494354	71.66	374	105	1	3.5E-97	350
gi|42545184	gi|37531422	57.21	444	157	33	4.4E-82	300
gi|1707308	gi|7450253	99.35	307	2	0	2.3E-82	300
gi|32415581	gi|109816	51.93	389	163	24	4.3E-67	250
gi|34597332	gi|28866546	50	440	202	18	5.3E-67	250
gi|4416379	gi|23003759	49.74	380	172	19	4E-52	200.3
gi|2822270	gi|115107	59.35	310	111	15	2.4E-52	200.3
gi|20877656	gi|18202616	71.73	428	107	14	5.7E-37	150.1
gi|151125	gi|48733237	86.1	374	50	2	4.5E-37	150.1
gi|2317687	gi|1395175	36.91	298	169	19	3.8E-22	100
gi|3122191	gi|6435584	45.08	193	104	2	2.2E-22	100
gi|18858297	gi|6563304	31.35	504	239	107	0.00000087	50
gi|115106	gi|7450252	25.65	269	182	18	0.00000041	50
