# Supplementary material for: Community detection in sequence similarity networks based on attribute clustering
Source: PLoS One. 2017 Jul 24;12(7):e0178650. doi: 10.1371/journal.pone.0178650 (PMC5524321; doi:10.1371/journal.pone.0178650)
Supplement: S1 Fig — (PDF) [file pone.0178650.s003.pdf]

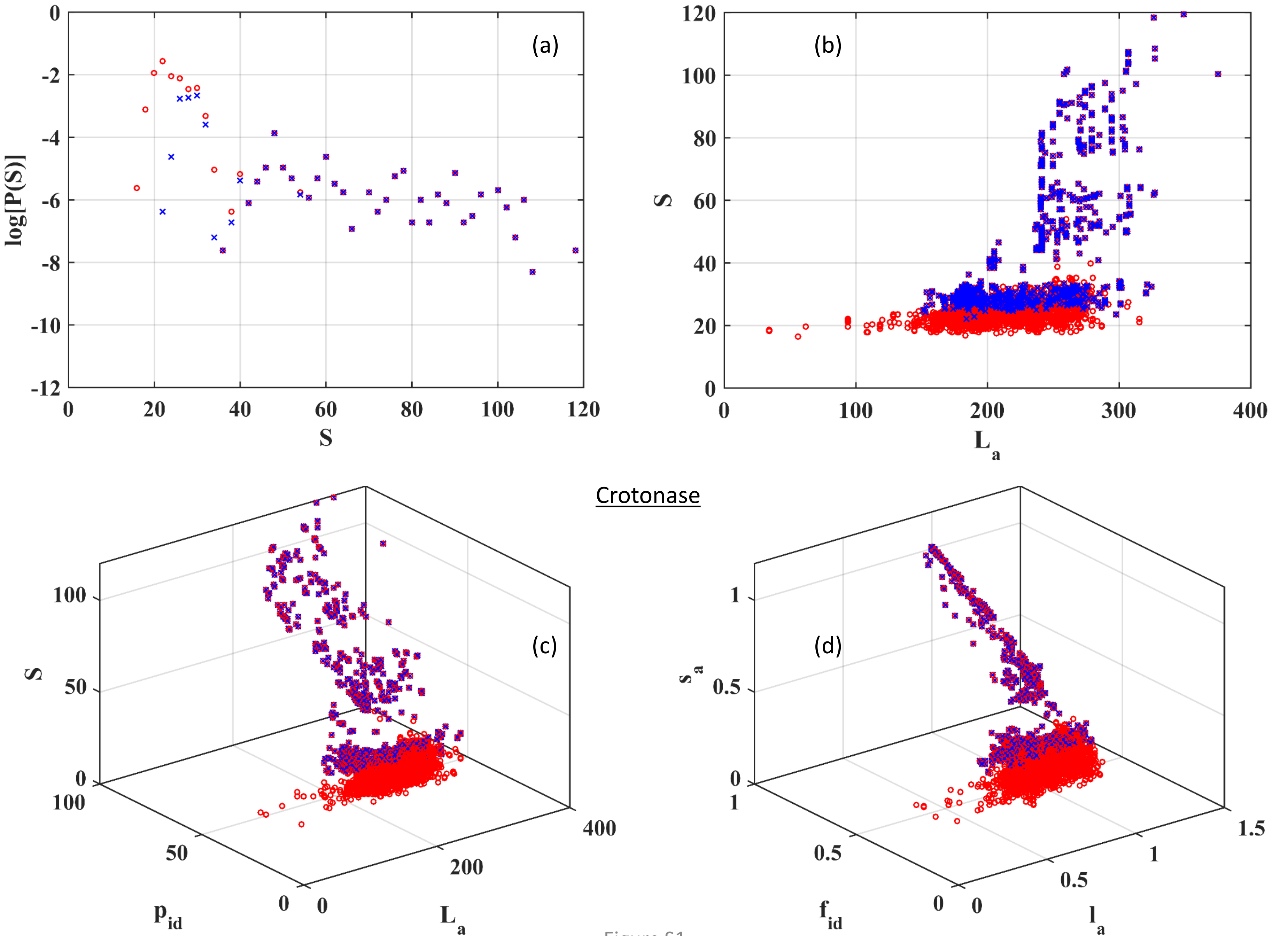

Figure S1

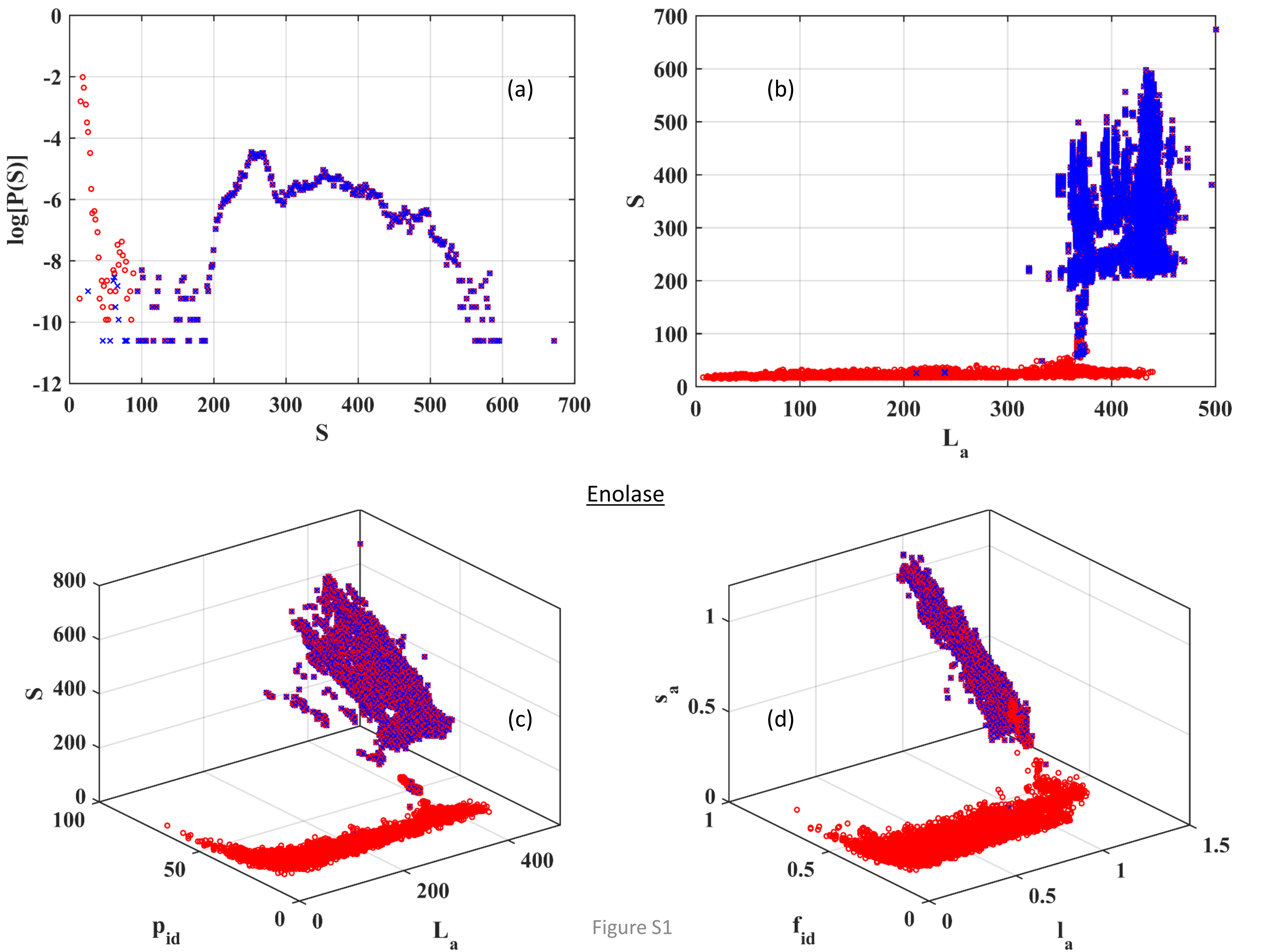

Figure S1

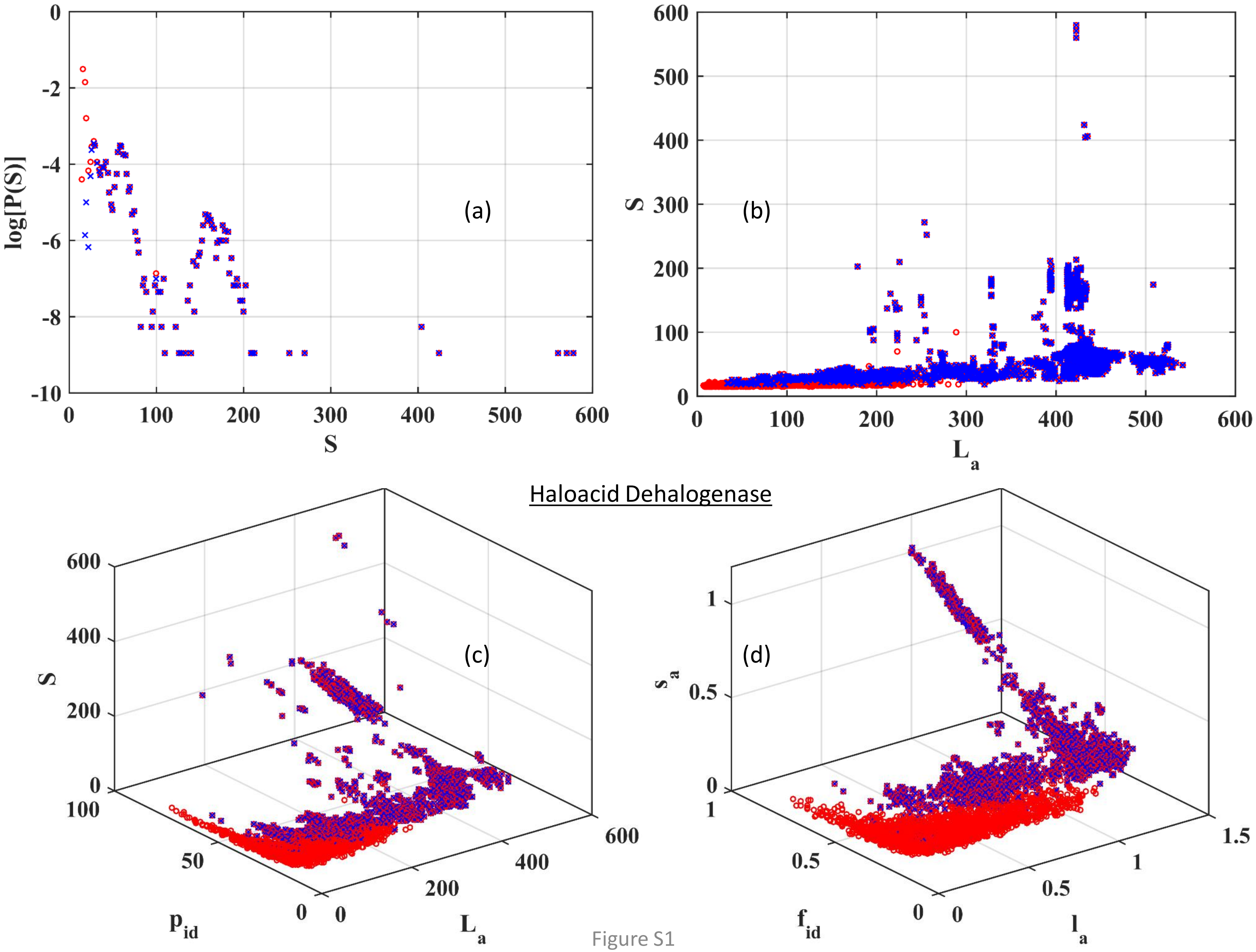

Figure S1

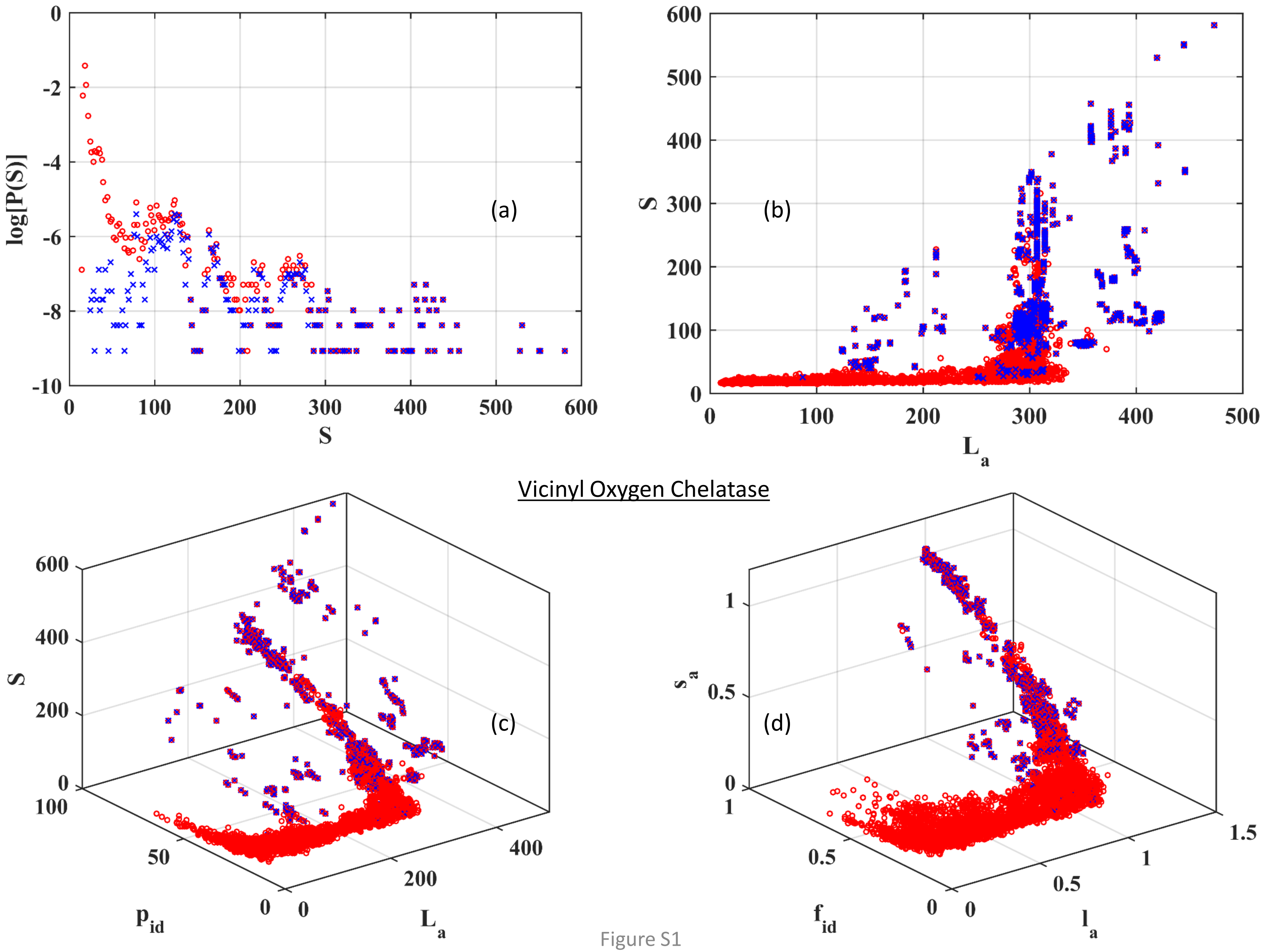

Figure S1

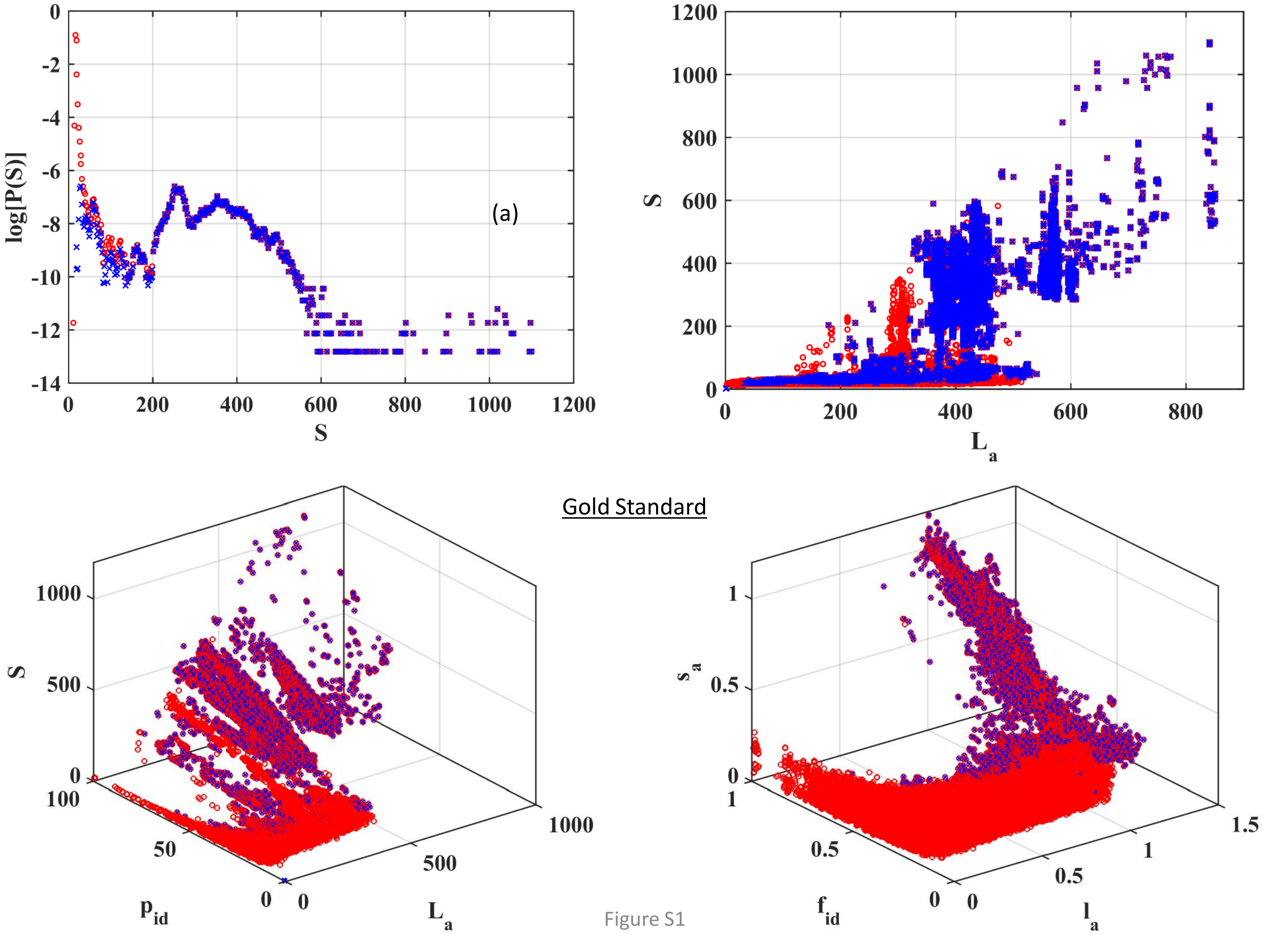

Gold Standard

Figure S1
