# Supplementary material for: Community detection in sequence similarity networks based on attribute clustering
Source: PLoS One. 2017 Jul 24;12(7):e0178650. doi: 10.1371/journal.pone.0178650 (PMC5524321; doi:10.1371/journal.pone.0178650)
Supplement: S2 Fig — (PDF) [file pone.0178650.s004.pdf]

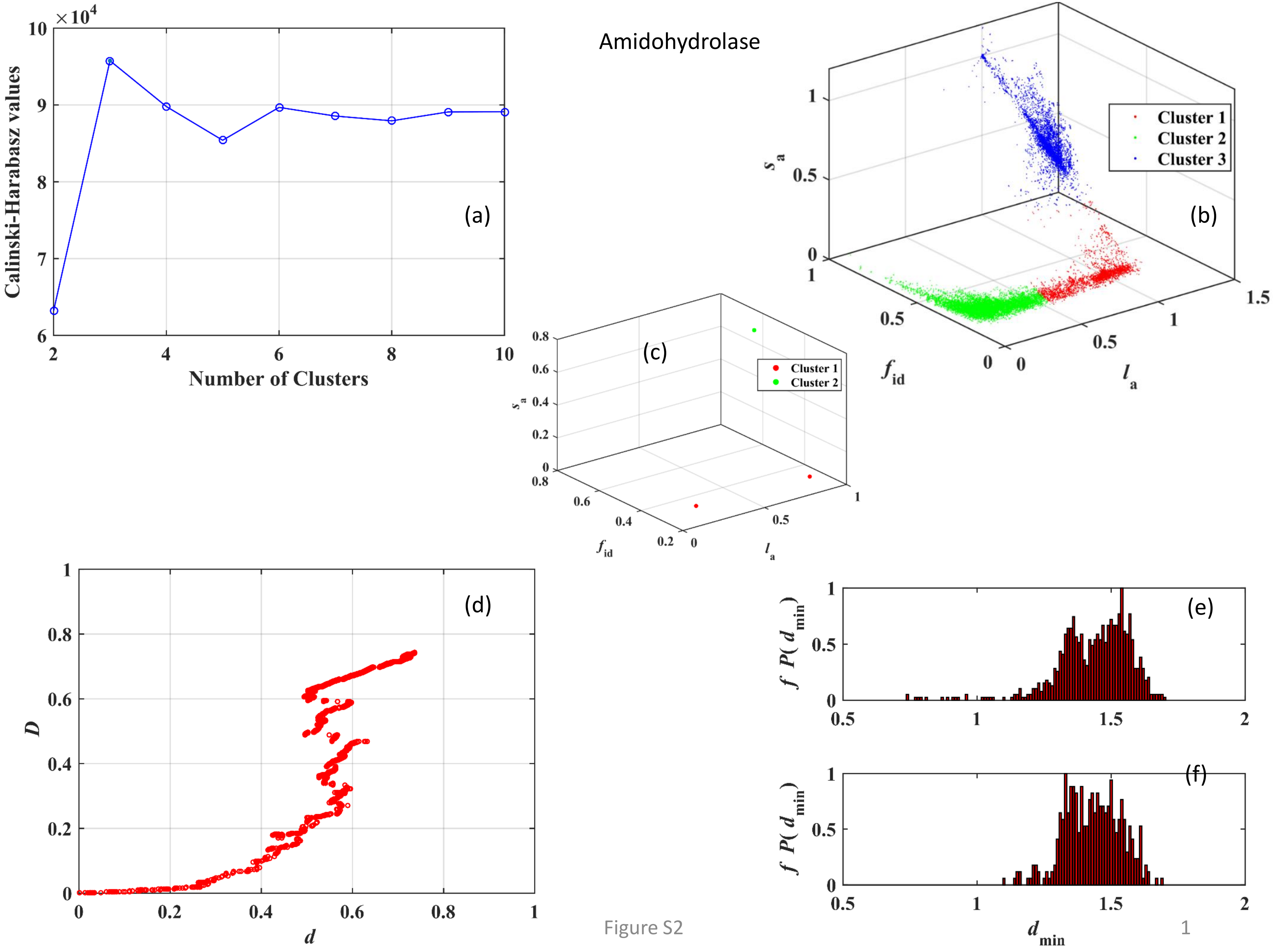
Amidohydrolase
(a)
Calinski-Harabasz values
×10^4
Number of Clusters
(b)
Cluster 1
Cluster 2
Cluster 3
s_a
f_id
l_a
(c)
Cluster 1
Cluster 2
(d)
D
d
(e)
(f)
f P(d_min)
d_min

Figure S2

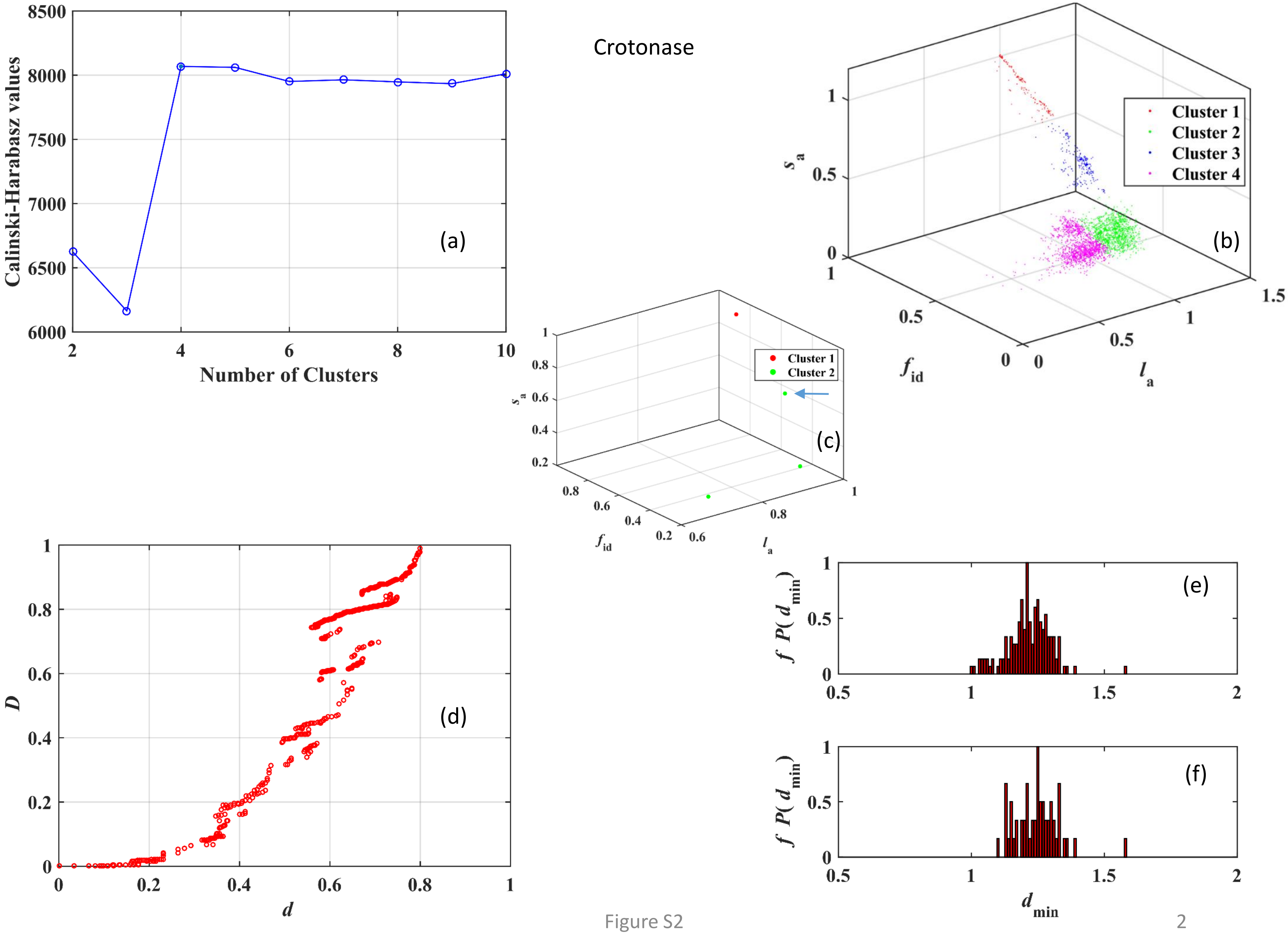
Crotonase
Calinski-Harabasz values
Number of Clusters
(a)
Cluster 1
Cluster 2
Cluster 3
Cluster 4
(b)
(c)
(d)
(e)
(f)
D
d
f P(d_min)
d_min

Figure S2

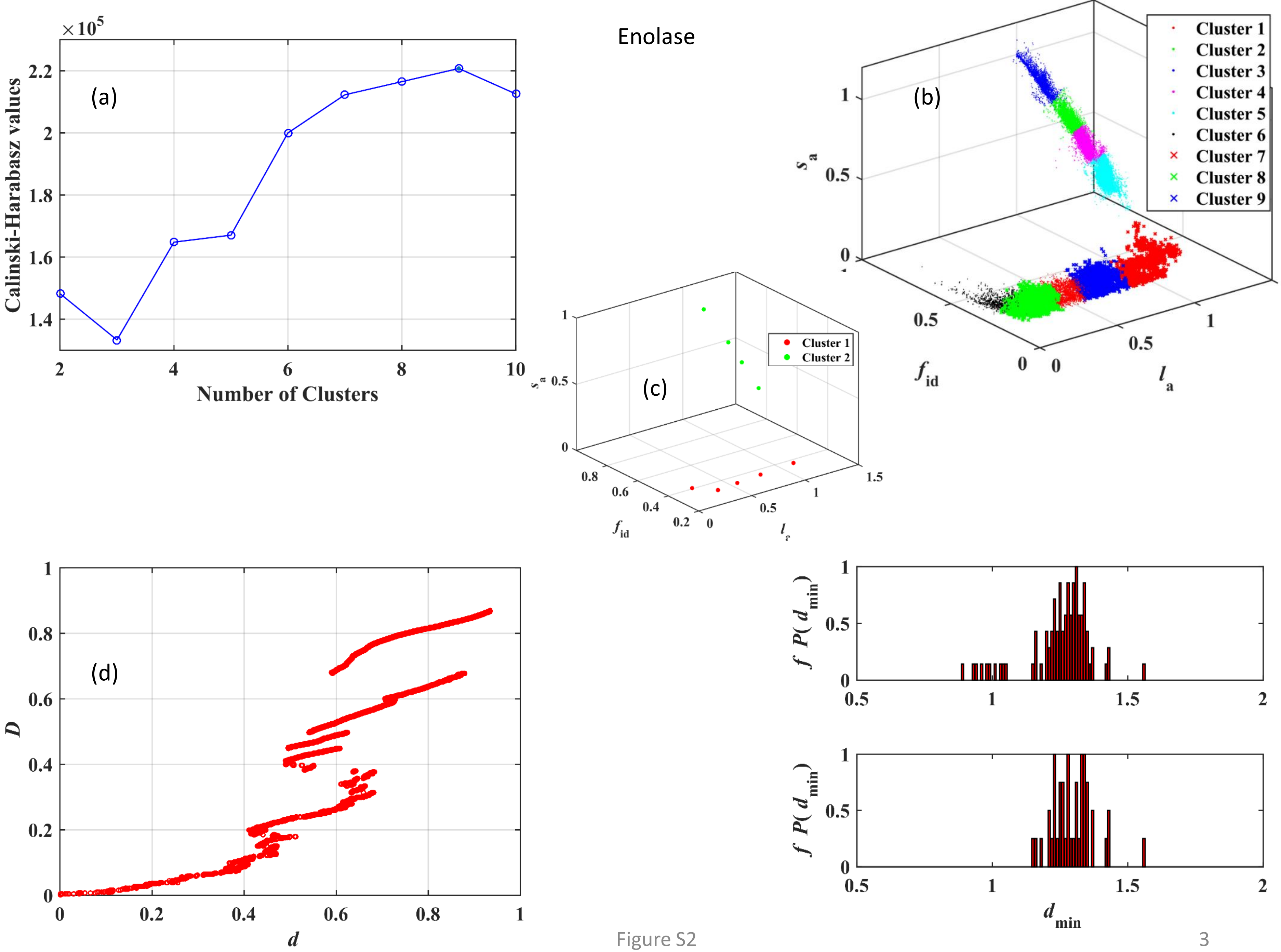
Enolase
(a)
×10^5
Calinski-Harabasz values
Number of Clusters
(b)
Cluster 1
Cluster 2
Cluster 3
Cluster 4
Cluster 5
Cluster 6
Cluster 7
Cluster 8
Cluster 9
s_a
f_id
l_a
(c)
Cluster 1
Cluster 2
(d)
D
d
f P(d_min)
d_min

Figure S2

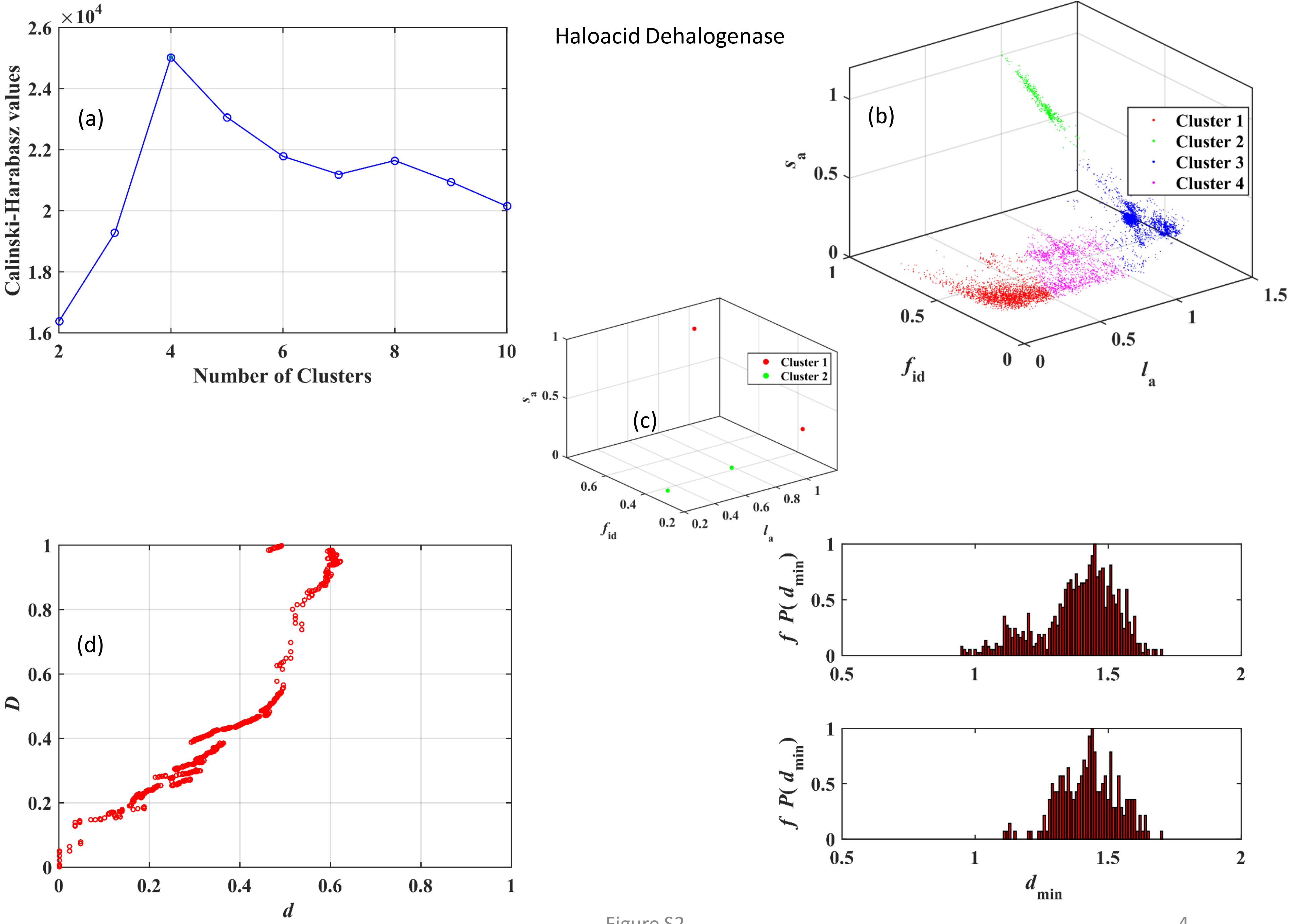
Haloacid Dehalogenase
(a)
Calinski-Harabasz values
×10^4
Number of Clusters
(b)
Cluster 1
Cluster 2
Cluster 3
Cluster 4
s a
f id
l a
(c)
Cluster 1
Cluster 2
s a
f id
l a
(d)
D
d
f P(d min)
d min

Figure S2

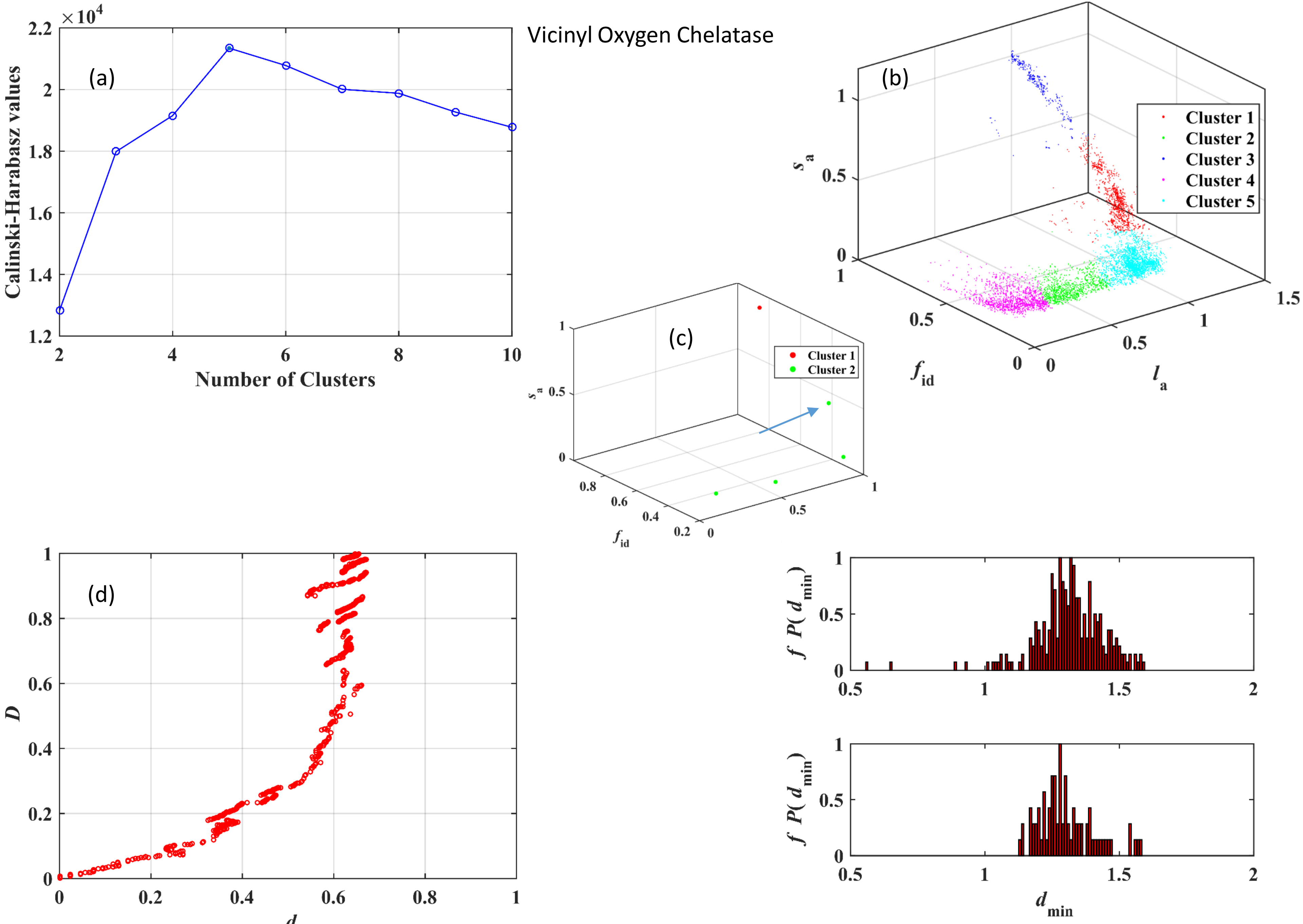
Vicinyl Oxygen Chelatase
(a)
Calinski-Harabasz values
×10^4
Number of Clusters
(b)
Cluster 1
Cluster 2
Cluster 3
Cluster 4
Cluster 5
s a
f id
l a
(c)
Cluster 1
Cluster 2
s a
f id
(d)
D
d
f P(d min)
d min

Figure S2

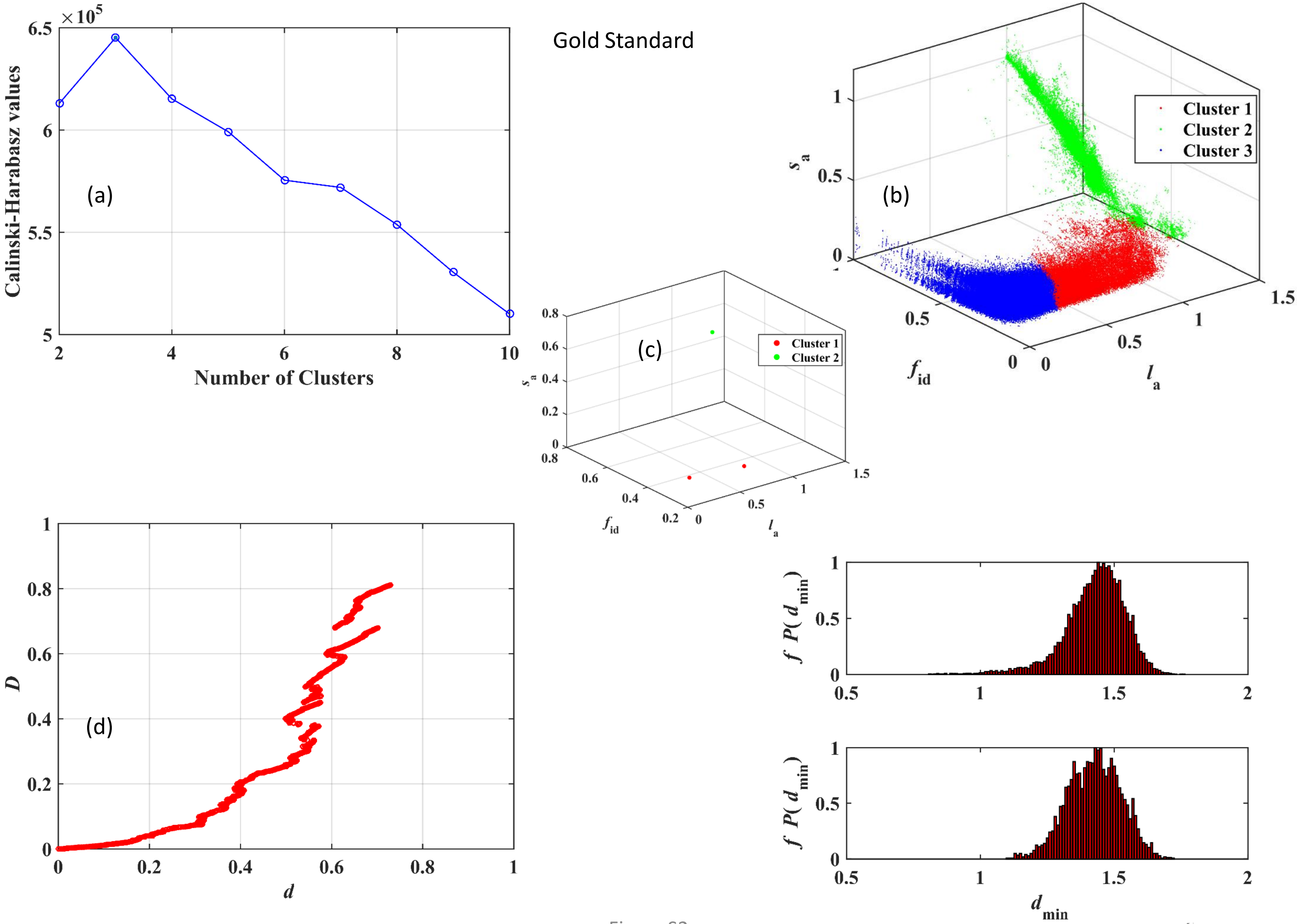
Gold Standard
(a)
Calinski-Harabasz values
×10^5
Number of Clusters
(b)
Cluster 1
Cluster 2
Cluster 3
s_a
f_{id}
l_a
(c)
Cluster 1
Cluster 2
(d)
D
d
$f\ P(d_{min})$
d_{min}

Figure S2
